# Observation of relativistic bond weakening in seaborgium hexacarbonyl

**DOI:** 10.1038/s41586-026-11105-2

**Published:** 2026-10-07

**Authors:** Alexander Yakushev, Jadambaa Khuyagbaatar, Christoph E. Düllmann, Valeria Pershina, Matthias Schädel, Jochen Ballof, Pavel Bartl, Michael Block, Raul-Andrei Cantemir, Dominik Dietzel, Francesca Giacoppo, Katharina Hermainski, Rolf-Dietmar Herzberg, Jan John, Jörg Krier, Nikolaus Kurz, Sven Löchner, Moumita Maiti, Pavol Mošať, Sebastian Raeder, Tetsuya K. Sato, Brigitta Schausten, Juha Uusitalo, Peter Wieczorek

**Affiliations:** 1 GSI Helmholtzzentrum für Schwerionenforschung GmbH, Darmstadt, Germany; 2 Helmholtz Institute Mainz, Mainz, Germany; 3 Johannes Gutenberg University Mainz, Mainz, Germany; 4 Czech Technical University in Prague, Prague, Czech Republic; 5 University of Liverpool, Liverpool, UK; 6 Indian Institute of Technology Roorkee, Roorkee, India; 7 Advanced Science Research Center, Japan Atomic Energy Agency, Tokai, Japan; 8 University of Jyväskylä, Jyväskylä, Finland

**Keywords:** Nuclear chemistry, Physical chemistry

## Abstract

The seventh row of the periodic table of the elements has been completed with the discovery of the five heaviest elements from flerovium (Fl, *Z* = 114) through oganesson (Og, *Z* = 118)^1^. Chemical properties of the superheavy elements (SHE, *Z* ≥ 104) are unique owing to the strong influence of relativistic effects on their valence electron shells^2^. The stability of the metal–ligand bonding in carbonyl complexes is a property that is predicted to be affected by such effects^3,4^. Here we present an experimental gas chromatography study on the stability of the Sg(CO)_6_ complex. The measured formation yields of Sg(CO)_6_ and W(CO)_6_ enabled determining the first bond dissociation energy (FBDE) of Sg(CO)_6_, which is 4(2) kJ mol^−1^ lower than that of W(CO)_6_ and corresponds to a value of 188(9) kJ mol^−1^. Thus, the trend of increasing FBDE values of hexacarbonyls within group 6 of the periodic table is reversed from W to Sg. Our result agrees with state-of-the-art theoretical calculations, which predict a decrease in the FBDE from W(CO)_6_ to Sg(CO)_6_ owing to the relativistic destabilization of the 6d atomic orbitals (AOs) in Sg (ref. ^4^). Also, the adsorption enthalpy value of Sg(CO)_6_ on gold of ${45}_{-6}^{+4}\,{\rm{kJ}}\,{{\rm{mol}}}^{-1}$ is reported.

## Abstract (web summary)

Gas chromatography analysis of compounds formed by the superheavy element seaborgium shows that the hexacarbonyl complex Sg(CO)_6_ exhibits lower first bond dissociation energy than the lighter chemical homologue W(CO)_6_, owing to relativistic bond weakening effects.

## Main

Man-made SHE (atomic number *Z* ≥ 104) are produced in nuclear fusion reactions by irradiating Pb, Bi or actinide targets with heavy ions. The rates of those reactions substantially decrease with increasing *Z*, reaching production rates of single atoms per week for Og. Also, extremely short lifetimes of SHE isotopes limit the number of chemical systems that can be studied experimentally^5–7^. SHE heavier than seaborgium (Sg, *Z* = 106) were chemically investigated only in the gas phase^8–15^.

Investigations of chemical bonding in compounds of the heaviest elements are of particular interest because strong relativistic effects influence their valence AOs. The 7s and 7p_1/2_ AOs are relativistically contracted and stabilized, leading to the closed shell (6d^10^7s^2^) and quasi-closed shell (7s^2^7p_1/2_^2^) configurations in copernicium (Cn, *Z* = 112) and flerovium (Fl, *Z* = 114), respectively, and thus to high volatility and inertness of these elements^8–12^. The stabilized 7p_1/2_ AO also leads to a weaker reactivity of nihonium (Nh, *Z* = 113) and moscovium (Mc, *Z* = 115) with respect to their lighter homologues Tl and Bi, respectively (ref. ^13^). In the elements of the 6d series, the valence 6d and 7s AOs participate in chemical bonding. The indirect relativistic effect is the destabilization and expansion of the 6d AOs, screened from the nucleus by the spherical inner s and p_1/2_ AOs. This effect is expected to influence properties of the 6d element compounds^2^, albeit to a lesser extent, as the central metal atom is surrounded by ligands^6^.

Among the known SHE compounds, the hexacarbonyl complex of Sg, Sg(CO)_6_, in which the Sg atom is in the zero-valent state and surrounded by six neutral carbon monoxide (CO) ligands, has attracted much attention^14^. In particular, the value of the FBDE, that is, the energy needed to detach one CO ligand from Sg(CO)_6_, is a measure of the compound stability and is expected to be influenced by relativistic effects^3,4^. Bonding in the hexacarbonyls of lighter homologues of Sg, that is, Cr, Mo, W, was originally investigated theoretically using the density functional theory (DFT) approach^16–18^. Two types of electron density transfer process were shown to be involved in the formation of the metal–CO bond: the σ-forth donation from the molecular orbital Φ_σ_(E_g_) of the CO ligands to the d(E_g_) AO of the metal atom (M) and the π-back donation from the d(T_2g_) AO of M to the Φ_π_(T_2g_) of CO (ref. ^16^). The influence of relativistic effects on the M–CO bond strength in hexacarbonyls was shown to increase from Cr to Mo and to W (ref. ^18^). Nash and Bursten calculated the FBDE of W(CO)_6_ and Sg(CO)_6_ using the relativistic effective core potentials (RECPs) and suggested that the FBDE value should increase from 46(2) kcal mol^−1^ for W(CO)_6_ to 47(2) kcal mol^−1^ for Sg(CO)_6_ owing to the stronger d(M) → (π)CO back donation overcompensating the weaker σ(CO) → d(M) donation from the CO ligands^3^. However, those calculations were unable to reproduce the experimental FBDE values for carbonyls of Mo and W and the FBDE of Sg(CO)_6_ has been obtained by normalizing the calculated values for the lighter complexes to the experimental ones^3^. The most recent FBDE calculations for Mo(CO)_6_, W(CO)_6_ and Sg(CO)_6_ were performed by Iliaš and Pershina^4^ using several non-relativistic and relativistic DFT approaches, as well as ab initio Dirac–Coulomb (DC) correlated methods, including  coupled cluster with single, double, and perturbative triple excitations (CCSD(T)). The best agreement with the experimental FBDE value of Mo(CO)_6_ and W(CO)_6_ was achieved by the Amsterdam Density Functional (ADF) spin–orbit (SO) and DC-DFT methods. These calculations for Sg(CO)_6_, at both the scalar relativistic (SR) and SO levels, predicted the FBDE of Sg(CO)_6_ to be about 10 kJ mol^−1^ smaller than that of W(CO)_6_. This decrease in the M–CO bond strength in Sg(CO)_6_ compared with W(CO)_6_, which is unexpected given the increasing trend from Cr to W, is ascribed to the reduction in the electron density transfer processes, that is, both in the σ-forth donation from the Φ_σ_(E_g_) of CO to the d(E_g_) AO of Sg and the π-back donation from the d(T_2g_) AO of Sg to the Φ_π_(T_2g_) of CO (ref. ^4^). The relativistic destabilization of the 6d(Sg) AOs is responsible for this effect (for details, see Methods section ‘Relativistic effects on the d(M) AOs’). The non-relativistic FBDE values were, by contrast, increasing from Mo to Sg, that is, Mo < W < Sg (ref. ^4^). These controversial predictions for the stability trend of the hexacarbonyls in group 6, along with a general desire to study the influence of relativistic effects on bonding in the 6d element compounds, sparked interest in related experimental studies.

The Sg carbonyl complex, Sg(CO)_6_, was synthesized in 2014 and its adsorption on a SiO_2_ surface was studied in comparison with hexacarbonyl complexes of Mo and W (ref. ^14^). Almost identical interaction strengths with the SiO_2_ surface, that is, adsorption enthalpy ($-\Delta {H}_{{\rm{ads}}}^{{\rm{Si}}{{\rm{O}}}_{2}}$) values, were reported for all three compounds^14^. Follow-up studies performed by different groups aimed at increasing the efficiency of such carbonyl complex studies^19–23^. These studies focused on determining the FBDE of the Sg hexacarbonyl complex and synthesis of carbonyl complexes of elements heavier than Sg. They also include the study of the chemical speciation of carbonyl compounds on a one-atom-at-a-time level performed using the laser ablation method in combination with time-of-flight mass spectrometry^23^. Before the present work, a first experimental attempt to measure the FBDE value for Sg(CO)_6_ was reported; however, no FBDE value for Sg(CO)_6_ has been given^24^.

## Observation of ^259^Sg carbonyl complexes

**Fig. 1:**
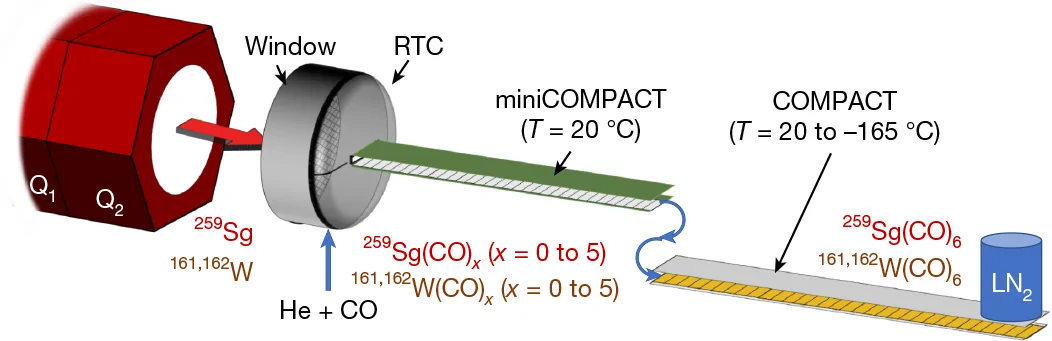
Experimental set-up behind the TASCA separator. Experimental set-up behind the second quadrupole magnet (Q_2_) at the end of the TASCA separator. Fusion products recoiling from the target, ^259^Sg (^161,162^W), produced in the nuclear reaction of the ^52^Cr ion beam with the ^208^Pb (^112^Sn) targets, were separated with TASCA and entered the RTC through a 6-μm-thick Mylar window. They were thermalized inside the RTC in a He + CO gas mixture and flushed out to the gas chromatography set-up. The first gas chromatography and detection channel, miniCOMPACT, is covered with a SiO_2_ layer and connected directly to the RTC exit. A negative temperature gradient was maintained along the second detector array, COMPACT, which has a Au layer on the detector surface. It was connected to miniCOMPACT with a 30-cm-long polytetrafluoroethylene capillary.

As described in Methods section ‘Experimental’, ^259^Sg nuclei were produced by means of the nuclear reaction ^208^Pb(^52^Cr,1n)^259^Sg (ref. ^25^) and isolated in the gas-filled separator TransActinide Separator and Chemistry Apparatus (TASCA). Nuclear reaction products were flushed from a gas-filled recoil transfer chamber (RTC), installed at the TASCA focal plane, into the combined gas chromatography and detection set-up. The latter consisted of two detector arrays, connected in series, in which the ^161,162^W and ^259^Sg species were deposited according to their volatility (see Fig. 1 and Methods section ‘Experimental’ for details). All products of the multistep process of the carbonyl complex formation and decomposition, which occur inside the RTC, are extracted as cations or neutrals by the gas flow into the first detection channel, miniCOMPACT^13^, except for those that adsorbed on the surface inside the RTC owing to finite extraction yields. The miniCOMPACT array, kept at room temperature, was directly connected to the RTC to detect non-volatile species, for example, unreacted atoms/ions and carbonyl complex fragments, M(CO)_*n*_ (1 ≤ *n* ≤ 5), extracted from the RTC by rapidly flowing gas and deposited in miniCOMPACT by diffusion-controlled deposition. The second array, COMPACT, was similar to that used in previous studies on the in situ formation and adsorption of W(CO)_6_ and Sg(CO)_6_ (ref. ^14^) but had a Au layer on the detector surface.

**Fig. 2:**
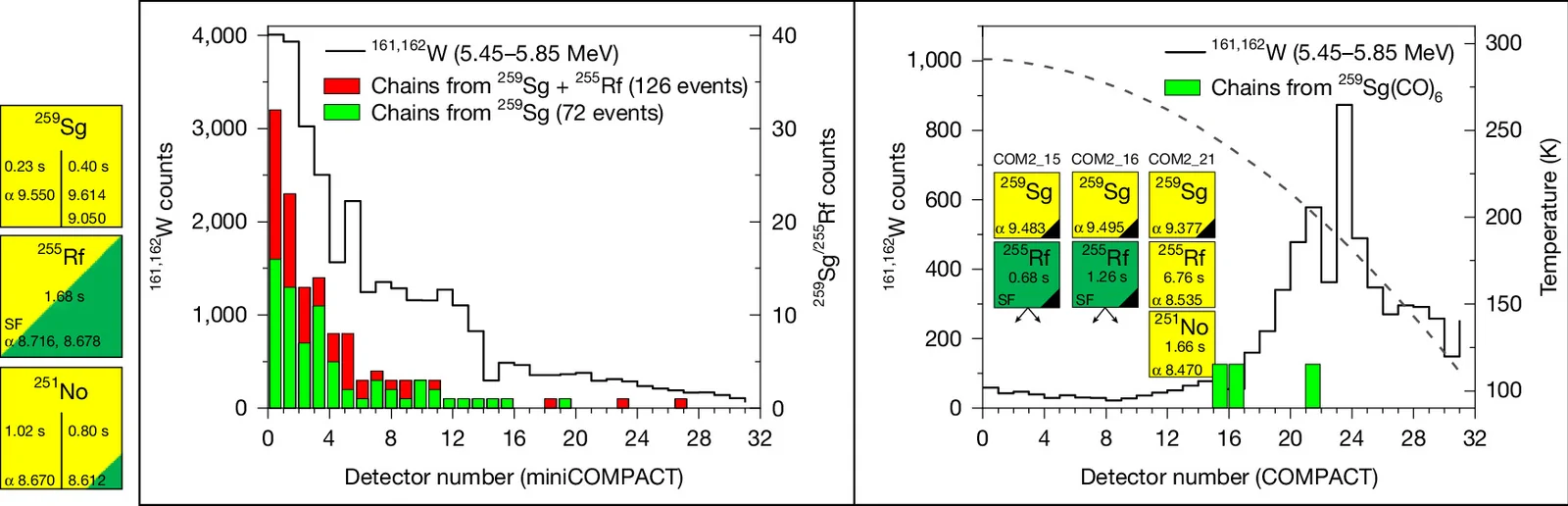
The observed event distributions from ^259^Sg and ^161,162^W in the detection set-up. The distributions of events registered in the miniCOMPACT detector array (left panel) and in the COMPACT detector array (right panel) originating from: ^259^Sg (green bars), sum of ^259^Sg and its daughter ^255^Rf (red bars) and ^161,162^W (black line histogram). The decay chains from ^259^Sg observed in COMPACT and their positions, as well as the temperature gradient in COMPACT (dashed grey line), are shown in the right panel, together with the experimental distribution pattern of ^161,162^W(CO)_6_. The known decay properties of the decay chain from ^259^Sg are shown to the left of the graph^27^. Colour coding in the decay chains: α decay (yellow) and spontaneous fission (SF) (green). The α-particle energies are given in MeV. The black triangles represent the decays observed during beam-off periods.

The presence of the ^161,162^W and ^259^Sg species inside the two arrays was determined by registration of their nuclear decay (see Methods section ‘Experimental’). As expected, the ^161,162^W and ^259^Sg species were found to be distributed between the two arrays. The species deposited in the first miniCOMPACT array (Fig. 2, left panel) were assigned to non-volatile metal atoms and carbonyl fragments M(CO)_*n*_ (1 ≤ *n* ≤ 5; M = ^161,162^W, ^259^Sg). These unsaturated carbonyl complex fragments are highly reactive and react very quickly with other molecules or surfaces they encounter^20,26^. Thus, on first contact with a solid surface in the first detector array, they readily adsorb on the SiO_2_ surface. The hexacarbonyl complexes, ^161,162^W(CO)_6_ and ^259^Sg(CO)_6_, known as volatile and non-reactive species, pass through the miniCOMPACT array and reach the COMPACT array, in which they adsorb on Au at lower temperatures according to their known volatility (Fig. 2, right panel).

In the miniCOMPACT array, 126 chains were detected. Of these, 72 chains were assigned to decay chains starting from ^259^Sg and 54 chains/spontaneous fission events to ^255^Rf, the α-decay daughter of ^259^Sg. Thus, about 40% of all chains were registered without the leading ^259^Sg decay, which is the result of the decay of short-lived ^259g^Sg (*T*_1/2_ = 402(56) ms) and ^259m^Sg (*T*_1/2_ = 226(27) ms)^27^ inside the RTC. The ratio between the total number of chains (126 events), starting from either ^259m,g^Sg or ^255^Rf, and the number of chains starting with the ^259m,g^Sg decay (72 events) allows estimating the flush-out time to about 250 ms. Three extra decay chains starting from ^259^Sg were observed in COMPACT and assigned to the deposition of ^259^Sg(CO)_6_. No further decay chains, with missing ^259^Sg, were registered in COMPACT. Only the events that started with ^259^Sg were used for the calculation of the ratio of chains between the two detectors. Using Poisson statistics for uncertainty quantification, the three observed Sg events in COMPACT translate to 1.43–6.27 events at the 68% confidence level^28^. About 20% of all ^161,162^W events were found in COMPACT forming a deposition peak of ^161,162^W(CO)_6_, whereas the main ^161,162^W fraction was found in miniCOMPACT, deposited as non-volatile species (Fig. 2). Thus, the ratio between volatile, M(CO)_6_, and non-volatile species, M(CO)_*n*_ (0 ≤ *n* ≤ 5) (M = ^161,162^W and ^259^Sg), registered in COMPACT and miniCOMPACT, respectively, was determined as 0.20 ± 0.01 for ^161,162^W and ${0.04}_{-0.02}^{+0.05}$ for ^259^Sg. These ratios indicate a rather low chemical yield of the hexacarbonyl complex formation. On the basis of Monte Carlo simulations^29^, the probability for nuclear decay in flight of volatile ^259m,g^Sg(CO)_6_ was estimated to be about 16%. Given the three detected events from ^259m,g^Sg(CO)_6_, this would add fewer than one event, which would be among the 72 ^259m,g^Sg events registered in miniCOMPACT. This would change the ratio of volatile and non-volatile for ^259m,g^Sg fractions to 0.05, which lies within the statistical error bars of the value ${0.04}_{-0.02}^{+0.05}$.

## Bond dissociation energy of ^259^Sg(CO)_6_

On thermalization in the carrier gas (He:CO = 1:1) inside the RTC, the ^259^Sg and ^161,162^W ions (or atoms) undergo collisions with He atoms and CO molecules, leading to subsequent chemical reactions, predominantly with neutral CO molecules. They can also react with gas impurities, for example, water molecules. Such adducts of metal ions (Ac^2+^ and No^2+^) with water and nitrogen molecules were recently observed behind the Berkeley Gas-filled Separator^30^. We kept the concentration of such impurities below about 1 ppm, so that formation of complexes with CO molecules was highly favoured over that of adduct formation.

Shorter-lived isotopes of Sg and W were used in the present study compared with the first study by Even et al.^14^, in which only volatile hexacarbonyl complexes could be registered. Still, the chemical yields of ^161,162^W(CO)_6_ and ^259^Sg(CO)_6_ observed in the present experiment are similar to the 10–20% for ^164^W and about 5% for ^265^Sg reported from the first study^24^. This allows concluding that the chemical equilibrium between carbonyl complex fragments can be reached on a very short timescale, as it was also observed in dissociation studies of the hexacarbonyl complexes of the lighter homologues with short-pulsed lasers^31^. Thus, the shorter half-lives of the Sg and W isotopes used in the present experiment did not lead to a reduction of the chemical yield.

Only the hexacarbonyls have an enhanced chemical inertness and volatility and can reach the COMPACT detector array. The experimental distribution of ^161,162^W(CO)_6_ and the position of the three ^259^Sg events in COMPACT were compared with deposition distributions obtained by a Monte Carlo simulation code based on the model of mobile adsorption^29^. Similarly low adsorption enthalpy values on the SiO_2_ surface for both W(CO)_6_ and Sg(CO)_6_, determined in the previous study^14^, allow for a rather simple ratio determination between the volatile (M(CO)_6_) and non-volatile (M(CO)_*n*_ (1 ≤ *n* ≤ 5; M = ^161,162^W, ^259^Sg) fractions. The analysis of the simulated distributions in both detector arrays suggests that the probability for the decay in flight of ^161^W and ^162^W in the miniCOMPACT is low and can be statistically neglected. Thus, the ratio between the sums of the ^161,161^W and ^259m,g^Sg events registered in COMPACT and miniCOMPACT, respectively, can be taken as the ratio between volatile and non-volatile fractions. The ratio between two W isotopes with different half-lives was determined by the analysis of the alpha energy spectrum from the first strip of the miniCOMPACT detector array as ^161^W:^162^W = 25:75. The Monte Carlo simulations of the distribution pattern were performed for the individual W isotopes. The final distribution for both isotopes, ^161^W and ^162^W, was obtained by weighted summation. For the simulation of the distribution pattern of ^259^Sg, an averaged half-life value of 320 ms was used, as obtained by Folden et al.^25^ in the experimental study using the same nuclear reaction ^52^Cr + ^208^Pb. This value is in agreement with the averaged half-life value for two states ^259m,g^Sg observed by Antalic et al.^27^.

**Fig. 3:**
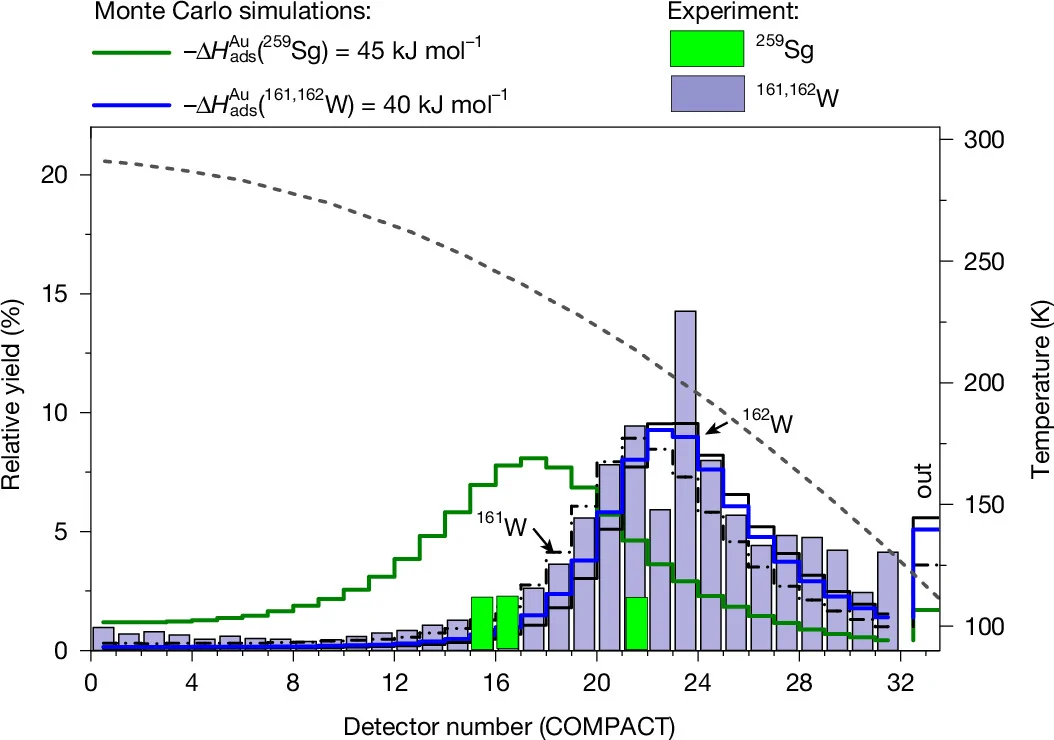
The experimental and simulated distributions of ^259^Sg and ^161,162^W in COMPACT. The experimentally determined position of ^161,162^W (blue bars) and ^259^Sg (green bars) hexacarbonyl molecules observed in the COMPACT detector array and their distributions obtained in Monte Carlo simulations (blue and green lines, respectively). The fractions of ^161,162^W(CO)_6_ and ^259^Sg(CO)_6_ in the simulated distributions, which are not deposited inside the detector array, are shown at the last position and marked as ‘out’. The dark grey dashed line represents the temperature gradient along the detector array.

The patterns of the experimental and simulated distributions of W(CO)_6_ and Sg(CO)_6_ on Au are shown in Fig. 3. The simulated ones, which describe the experimental distributions well, correspond to their adsorption enthalpy values, $-\Delta {H}_{{\rm{ads}}}^{{\rm{Au}}}({\rm{W}}{({\rm{CO}})}_{6})=40(5)\,{\rm{kJ}}\,{{\rm{mol}}}^{-1}$ and $-\Delta {H}_{{\rm{ads}}}^{{\rm{Au}}}({\rm{Sg}}{({\rm{CO}})}_{6})={45}_{-6}^{+4}\,\,{\rm{kJ}}\,{{\rm{mol}}}^{-1}$, respectively. The error bars were evaluated on the basis of statistical considerations—the simulated distributions at the given limits reproduce the experimental ones at the 68% confidence level. In the present study, the COMPACT detector surface was covered with Au, in contrast to the first experiment with Sg(CO)_6_, in which a SiO_2_-covered COMPACT detector array was used^14^. The $-\Delta {H}_{{\rm{ads}}}^{{\rm{Au}}}$ value of W(CO)_6_ on the Au surface determined in the present experiment is close to that from previous studies on Au, $-\Delta {H}_{{\rm{ads}}}^{{\rm{Au}}}({\rm{W}}{({\rm{CO}})}_{6})={41}_{-4}^{+3}\,{\rm{kJ}}\,{{\rm{mol}}}^{-1}$, obtained by Even et al.^32^. Notably, we find that the $-\Delta {H}_{{\rm{ads}}}^{{\rm{Au}}}$ values of Sg(CO)_6_ and W(CO)_6_ on Au are several kJ mol^−1^ lower than the $-\Delta {H}_{{\rm{ads}}}^{{\rm{Si}}{{\rm{O}}}_{2}}$ values on a SiO_2_ surface^14,33^. The values indicate that the deposition of volatile hexacarbonyl complexes well below room temperature on both surfaces occurs owing to physisorption. However, the interaction with the surface can be potentially influenced, for example, by CO molecules adsorbed on the cold Au surface^34^ or by the level of hydroxylation of the SiO_2_ surface^35^.

In the multistep decomposition–recombination process, the rates of the recombination reactions with CO ligands are close to zero under vacuum conditions and reach equilibrated values at a CO gas pressure of several tens of millibars^36^. The carbonyl complex formation in the present study was favoured by a high CO partial pressure. However, the decomposition process is facilitated by intense fluences of neutrons, electrons and photons as well as fast He ions from the He filling gas^37^. Thus, chemical interactions between the Sg and W ions or atoms with CO molecules inside the RTC are multistep decomposition and recombination processes, which result in a variety of metal–carbonyl species in a dynamic equilibrium.

The relation between the rate constant and the FBDE was commonly applied in the decomposition studies of carbonyl complexes. This method, described in Methods section ‘Determining the stability of the complex’, was used for the determination of FBDE values of several carbonyl complexes relative to the known FBDE value of the reference complex, Cr(CO)_6_ (ref. ^38^). Applying this method, the measurement of the ^161,162^W(CO)_6_ and ^259^Sg (CO)_6_ fractions under identical conditions allows for the direct determination of the FBDE value for Sg(CO)_6_ relative to the known value for W(CO)_6_. The formation of the carbonyl complexes of ^161,162^W and ^259^Sg was studied under identical conditions, that is, equal reaction times and CO concentration. Furthermore, the concentrations of all metal carbonyl species are negligibly small compared with the concentration of the other reactant, CO gas. Therefore, the ratio between the concentration of ^161,162^W(CO)_6_ and ^259^Sg(CO)_6_ can be described as the ratio of their rate constants (see Methods section ‘Determining the stability of the complex’ for details):

1 $$\frac{\left[\genfrac{}{}{0ex}{}{161,162}{}{\rm{W}}{({\rm{CO}})}_{6}\right]}{\left[\genfrac{}{}{0ex}{}{259}{}{\rm{Sg}}{({\rm{CO}})}_{6}\right]}=\frac{{k}_{{\rm{W}}}}{{k}_{{\rm{Sg}}}}=\frac{{A}_{{\rm{W}}}{{\rm{e}}}^{\frac{{{\rm{FBDE}}}_{{\rm{W}}}}{{\rm{RT}}}}}{{A}_{{\rm{Sg}}}{{\rm{e}}}^{\frac{{{\rm{FBDE}}}_{{\rm{Sg}}}}{{\rm{RT}}}}},$$

in which *A*_W_ and *A*_Sg_ are values of the Arrhenius pre-factor for W(CO)_6_ and Sg(CO)_6_, respectively. In our study, we used the value for the Arrhenius pre-factor of 10^15.6^ for both hexacarbonyl complexes, which was used also for Mo and W hexacarbonyls in ref. ^38^. A smaller Sg(CO)_6_ fraction compared with W(CO)_6_ should result in a lower FBDE value of the Sg(CO)_6_ complex. Using the obtained ratios of volatile and non-volatile fractions of 0.20(1) for ^161,162^W and ${0.04}_{-0.02}^{+0.05}$ for ^259^Sg, an energy difference of 4(2) kJ mol^−1^ between the FBDE values of W(CO)_6_ and Sg(CO)_6_ was calculated. By subtracting this difference from the experimental FBDE value of W(CO)_6_ of 192(8) kJ mol^−1^ (ref. ^38^), the FBDE value for Sg(CO)_6_ results in 188(9) kJ mol^−1^. The error bar was obtained by mean squared summing and is dominated by the large error of the experimental FBDE value for W(CO)_6_. A more precise determination of this value would reduce the error bar of the value of Sg(CO)_6_.

**Fig. 4:**
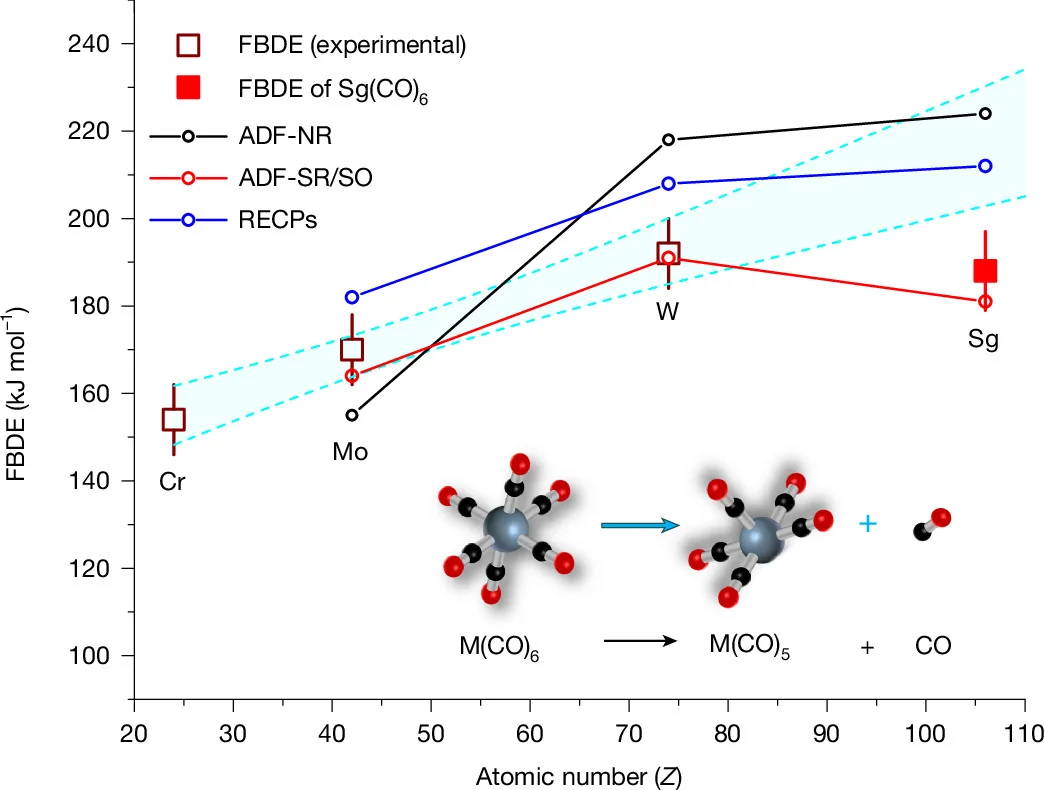
The experimental and calculated FBDE values of the group 6 carbonyl complexes. The experimental FBDE values of the carbonyl complexes M(CO)_6_ (M = Cr, Mo, W) from ref. ^38^ are shown by open brown squares. Error bars, derived from ref. ^38^ and this work, are represented as vertical lines. The cyan-coloured area inside the dashed lines shows the 85% prediction interval assuming a linear relation between the experimental FBDE values and atomic number (*Z*). The FBDE value of Sg(CO)_6_, obtained in the present study, is shown as a filled red square. Theoretical FBDE values, predicted for Mo(CO)_6_, W(CO)_6_ and Sg(CO)_6_ by calculations using different methods, are shown with black (ADF-NR)^4^, blue (relativistic RECPs-CCSD(T))^3^ and red (ADF-SR/SO)^4^ open circles.

Figure 4 shows the experimental FBDE value obtained for Sg(CO)_6_ along with those for the lighter homologues from ref. ^38^, in comparison with the theoretical data obtained at the different levels of theory^3,4^. We can see that the calculations using the non-relativistic ADF method (ADF-NR) predict an increasing trend in FBDE from Mo to W and Sg (ref. ^4^). The calculations using the RECPs-CCSD(T) method also give a 4 kJ mol^−1^ higher FBDE value for Sg(CO)_6_ compared with that of W (ref. ^3^). However, those calculations were unable to reproduce the experimental FBDE values for lighter homologues (Fig. 4). Our experimental data revealed a 4(2) kJ mol^−1^ decrease in the FBDE from W(CO)_6_ to Sg(CO)_6_, being smaller than that predicted by Iliaš and Pershina^4^ (about 10 kJ mol^−1^ using the ADF-SO and DC-DFT methods and 15 kJ mol^−1^ using the X2c-CCSD(T) method), however, in full agreement about the decreasing trend.

## Conclusions

The stability of the Sg(CO)_6_ complex was investigated in the present work. This was done by a comparative study of carbonyl complex formation with the short-lived isotopes ^161,162^W and ^259^Sg on a single-atom-at-a-time level. The formation of carbonyl complexes was performed in situ on thermalization in a He + CO gas mixture. Products of the multistep chemical interactions, the non-volatile metal–carbonyl fragments and the volatile hexacarbonyl complexes ^161,162^W(CO)_6_ and ^259^Sg(CO)_6_, were detected individually in two detector arrays. Three out of 75 registered events from ^259^Sg were found in the second detector array and were assigned to the ^259^Sg(CO)_6_ complex. This allowed for determining the fractional yields of the hexacarbonyl complexes and a ratio of the rate constants for the last step of the hexacarbonyl formation. On this basis, the FBDE of Sg(CO)_6_ was found to be 4(2) kJ mol^−1^ lower than that of W(CO)_6_. This trend reversal in the increasing FBDE values of the group 6 hexacarbonyls from W to Sg is in agreement with the results of the latest relativistic calculations^4^. According to these calculations, the predicted Sg–CO bond weakening is a result of a decrease in both the σ-forth and π-back bonding in Sg(CO)_6_ caused by the relativistic destabilization of the 6d(Sg) AOs (ref. ^4^). Thus, the FBDE of a SHE compound, Sg(CO)_6_, was measured for the first time and is found to be in excellent agreement with modern theory. Our study presents a sensitive method for investigating the influence of relativistic effects and opens a new pathway for determining physical observables in the chemistry of the heaviest elements.

## Methods

### Experimental

The synthesis of ^259^Sg(CO)_6_ and ^161,162^W(CO)_6_ was performed at the gas-filled separator TASCA at the GSI Helmholtzzentrum für Schwerionenforschung GmbH, Darmstadt, Germany^39^, and was similar to that in the first study of the ^265^Sg(CO)_6_ carbonyl complex at the RIKEN Nishina Center for Accelerator-Based Science, Wako, Japan^14^. At RIKEN, the isotope ^265^Sg was produced by irradiating ^248^Cm targets with a ^22^Ne beam, separated with the gas-filled recoil separator GARIS. Only volatile hexacarbonyl complexes Mo(CO)_6_, W(CO)_6_ and Sg(CO)_6_ were transported through a 10-m-long Teflon capillary to a SiO_2_-covered chromatography column. Here a negative temperature gradient was applied^14^. This column also served as a radiation detector for the identification of the studied radionuclides. In separate experiments, the behaviour of Mo and W carbonyl compounds was also studied.

In the present work, the isotope ^259^Sg, which has two states, ^259g^Sg (*T*_1/2_ = 402(56) ms) and ^259m^Sg (*T*_1/2_ = 226(27) ms)^27^, was produced in the cold nuclear fusion reaction ^208^Pb(^52^Cr,1n)^259^Sg (ref. ^25^). Short-lived radioisotopes of W, a chemical homologue of Sg, were produced by the nuclear reaction ^112^Sn(^52^Cr; 2-3*n*)^161,162^W (*T*_1/2_(^161^W) = 409 ms; *T*_1/2_(^162^W) = 1.19 s). ^52^Cr^2+^ ions, provided from a Penning ionization gauge (PIG) ion source, were accelerated by the UNIversal Linear ACcelerator (UNILAC) at the GSI Darmstadt to energies of 4.95(2) MeV u^−^^1^. A pulsed (5 ms beam-on, 15 ms beam-off) ^52^Cr^14+^ ion beam (average beam intensity of 6 × 10^12^ per second) irradiated the ^112^Sn and ^208^Pb targets. Targets were made from ^208^PbS and ^112^SnO_2_ and mounted on a rotating target wheel^40^. The target material was thermally evaporated onto carbon foils with an average thickness of 100(5) μg cm^−^^2^. Each target wheel contained four segments of ^208^PbS or ^112^SnO_2_ with an average thickness of 0.80(5) and 0.60(5) mg cm^−^^2^, respectively. In total, 4.0(2) × 10^18^ ^52^Cr^14+^ ions impinged on the ^208^Pb targets during the two-week experimental Sg run. The W isotopes were produced in a 2-h run, during which a beam integral of 4.4(1) × 10^16^ was collected.

The nuclear fusion–evaporation products were separated from the intense primary beam and from unwanted products of multi-nucleon transfer reactions with the gas-filled recoil separator TASCA. TASCA, consisting of one dipole (D) and two quadrupole (Q) magnets in a DQ_1_Q_2_ configuration, was operated in the high transmission mode^39^. A He filling gas at a pressure of *p*_He_ = 0.8 mbar was used in TASCA. The ^259^Sg and ^161,162^W ions, recoiling from the target, were guided through TASCA, in which the magnets were set to rigidities of 2.16 and 1.64 Tm for Sg and W, respectively.

The formation process and volatility of carbonyl complexes were investigated by applying a gas–solid chromatography method^5,6^. Adsorption studies were conducted on two surface types, SiO_2_ and Au, in the temperature range from room temperature to *T* = 110 K. Evaporation residues reaching the RTC (diameter: 60 mm, depth: 20 mm) were thermalized in a 1:1 gas mixture of He and CO gas at 1 bar (Fig. 1), similarly to the first Sg(CO)_6_ study performed at RIKEN, Japan^14^. The gas chromatography and detection set-up consisted of two detector arrays in series and was similar to the set-up used for the chemistry studies with Nh and Mc (ref. ^13^). The first array (miniCOMPACT) was 16 cm long and was directly attached to the RTC exit. The second array (COMPACT) was 32 cm long and was connected to the first detector array by a 30-cm-long polytetrafluoroethylene capillary with an inner diameter of 2 mm. Both detector arrays consist of two panels facing each other, each divided into 32 positive–intrinsic–negative diodes mounted in a row. These radiation detectors registered α particles and fission fragments emitted by nuclear decays occurring inside the detector channel. The distance between opposite detector panels is 0.6 mm, thus, they form a narrow gas channel with a cross-section of 10.0 × 0.6 mm^2^. The silicon photodiodes were covered with a 30–50-nm-thick layer of SiO_2_ (miniCOMPACT) or Au (COMPACT)^10,12,13^. The detector pair 15 of miniCOMPACT and the bottom strip 23 of COMPACT lost functionality during the experiment. The gas mixture of 50% He (99.9999%) and 50% CO (99.997%) circulated in a closed loop at a flow rate of 2.5 l min^−1^. Less than 100 ms are required to flush out the whole detection set-up. The gases were further purified of water impurities using MC400-202FV (SAES) cartridges installed in a closed loop. These cartridges had internal particle filters that efficiently removed aerosol particles down to a size of 3 nm. The water content in the carrier gas was on a level of about 1 ppm.

### Determining the stability of the complex

It is known that the decomposition products of the hexacarbonyl complexes, that is, the residual fragments, formed by means of removal of one (or more) CO ligand(s) on contact with a hot metal surface, are substantially less volatile and more reactive than the hexacarbonyls^41^. The thermal decomposition of hexacarbonyl complexes on a metal surface was proposed as a method of choice to determine carbonyl compound stabilities on a one-atom-at-a-time level^20,26^. For this, in situ-formed volatile hexacarbonyl complexes are transported by a gas flow through an isothermal column made of metallic silver, placed upstream of a detector array and kept at elevated temperatures. Thus, hexacarbonyl complexes decomposing on the metal surface do not reach the detector. The breakthrough yield through the decomposition column measured at different temperatures is indicative of the stability of the hexacarbonyl complex—a higher stability, that is, FBDE, translates to a higher temperature, at which a certain fraction, for example, 50%, decomposes inside the column^20^. This approach was validated using Monte Carlo simulations for the evaluation of the FBDE of Mo(CO)_6_ and W(CO)_6_ and laid a basis for the expected behaviour of Sg(CO)_6_ (ref. ^26^). However, an earlier attempt to determine the FBDE value of ^265^Sg(CO)_6_ was unsuccessful, probably because of limited statistics, because a much larger number of Sg atoms is required to measure the breakthrough curve in the isothermal regime^24^.

In macrochemistry, the stability of carbonyl complexes with 3d, 4d and 5d transition metals is commonly studied by infrared laser absorption spectroscopy or by mass spectrometry of the fragments following the dissociation of the complexes induced by thermal dissociation, photo-dissociation or particle impact^42–44^. These studies showed the relation between the fragmentation pattern and individual bond energies. Mass spectra of positively charged products of the M(CO)_6_ complex dissociation (M = Cr, Mo, W) show the existence of all M(CO)_*n*_ (0 ≤ *n* ≤ 6) fragments, from metallic ions up to hexacarbonyl ions, even after interactions at low energy and for very short (several microseconds) interaction times^31,45^.

**Extended Data Fig. 1:**
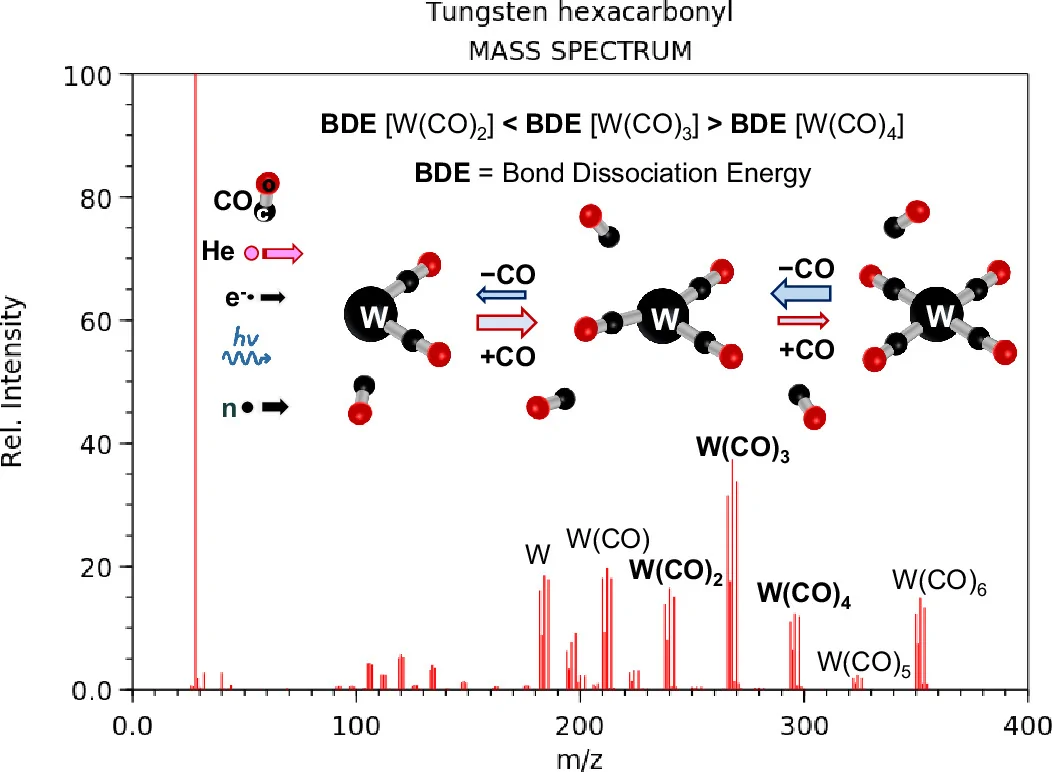
The mass spectrum of W(CO)_6_ observed by its decomposition. A scheme of decomposition–recombination processes inside the RTC illustrates different abundances of W(CO)_*x*_ (*x* = 2 to 4) ions in the mass spectrum of W(CO)_6_, which is shown as the red histogram^51^. The arrows represent the rates of decomposition (blue) or recombination (red) processes; larger arrows correspond to larger rate constants.

The mass spectra obtained by electron impact decomposition include all fragments already at low electron energies. From roughly 70 eV on, the fragment ratios become fully equilibrated^43^. The measurement of the fragmentation pattern and of ionization energy thresholds (appearance energies) for all fragments allows for evaluating the bonding energy between the metal atom and the last bound CO ligand for the individual fragments with different numbers of ligands^43^. These branching ratios carry substantial information on the thermochemistry and photodissociation dynamics of metal carbonyls and allow for the unambiguous determination of the individual CO bond dissociation energies^46^. The abundances of individual fragments in the equilibrated state reflect their thermochemical and kinetic behaviour (Extended Data Fig. 1). Thus, the individual CO bond dissociation energies can be determined from a measurement of the individual fragment concentrations^46^.

All of the separation processes of CO ligands from carbonyl complexes and their fragments are concurrently accompanied by recombination processes, in which a free CO molecule attaches back to a carbonyl fragment, especially at non-negligible CO partial pressure^47^. Summarizing the previous studies on the decomposition of the hexacarbonyl complexes, we conclude that the decomposition is a multistep and reversible process, in which the equilibrium is reached on a microsecond timescale. Thus, the method, which is based on the determination of fractional yields and their relationship to the corresponding binding energies, can also be applied to carbonyl complexes produced at the single-atom level.

The fractional yields depend on the rate constants of the individual decomposition/recombination steps and, therefore, on the activation energy for that specific reaction step. The rate constant for a selected decomposition step

2 $${\rm{M}}{({\rm{CO}})}_{n}\mathop{\to }\limits^{{k}_{n}}{\rm{M}}{({\rm{CO}})}_{n-1}+{\rm{CO}}$$

is *k*_*n*_ and equal to the negative rate constant of the reverse recombination step −*k*_*n*_,

3 $${\rm{M}}{({\rm{CO}})}_{n-1}+{\rm{CO}}\mathop{\to }\limits^{-{k}_{n}}{\rm{M}}{({\rm{CO}})}_{n}$$

According to the Arrhenius equation, the value of the rate constant depends on the pre-exponential frequency factor (*A*), the activation energy of the reaction (*E*_a_) and the temperature (*T*) as

4 $$k=A{{\rm{e}}}^{\frac{-{E}_{{\rm{a}}}}{{RT}}},$$

in which *R* is the universal gas constant. The activation energy for a certain decomposition or recombination step is equal to the bonding energy for that specific CO ligand with the central metal atom. In the last step of the hexacarbonyl formation, M(CO)_5_ + CO ↔ M(CO)_6_, that is, the first step of the hexacarbonyl decomposition, which is especially interesting for the present study, the rate constant depends on the value of the FBDE, which replaces the activation energy *E*_a_ in equation (4) for the first decomposition step^26^.

The relation between the rate constant and the FBDE was commonly applied in the decomposition studies of carbonyl complexes. Therefore, the decrease of the M(CO)_6_ concentration in the laser-induced decomposition was determined as a product [M(CO)_6_] = *kτ*_r_, in which *k* is the rate constant and *τ*_r_ is the reaction time^38,48^. The method described here was applied to determine the experimental FBDE values in carbonyl complexes with Mo, W and Fe, relative to the Cr carbonyl complex, measured by laser-induced photodissociation. In group 6, the FBDE values of hexacarbonyl complexes, and, thus, the stability, increased from Cr to Mo and further to W (ref. ^38^).

In the present work, the formation of the carbonyl complexes of ^161,162^W and ^259^Sg was studied under identical conditions, that is, equal reaction times and CO concentration, similarly to decomposition studies with lighter homologues, Cr, Mo and W (refs. ^38,48^). Furthermore, the concentrations of all metal carbonyl species are negligibly small compared with the concentration of the other reactant, the CO gas. Therefore, the measured M(CO)_6_ fractions can also be determined as a product [M(CO)_6_] = *kτ*_r_ (M = ^161,162^W and ^259^Sg) and the ratio between the concentration of ^161,162^W(CO)_6_ and ^259^Sg(CO)_6_ can be described as the ratio of their rate constants:

5 $$\frac{\left[\genfrac{}{}{0ex}{}{161,162}{}{{\rm{W}}({\rm{CO}})}_{6}\right]}{\left[\genfrac{}{}{0ex}{}{259}{}{{\rm{Sg}}({\rm{CO}})}_{6}\right]}=\frac{{k}_{{\rm{W}}}{\tau }_{{\rm{r}}}}{{k}_{{\rm{Sg}}}{\tau }_{{\rm{r}}}}=\frac{{k}_{{\rm{W}}}}{{k}_{{\rm{Sg}}}},$$

Combining equations (4) and (5), we can conclude that the measured ratio between the concentration of ^161,162^W(CO)_6_ and ^259^Sg(CO)_6_ yields the difference between the corresponding FBDE values.

Applying the proposed method and determining fractional yields of specific carbonyl complexes by a mass analysis method, bond dissociation energies can be determined for individual carbonyl fragments identified in future experiments on the single-atom level. The chemical speciation is especially important for carbonyl complexes with odd-*Z* elements, in which, unlike in macroscopic amounts, only mononuclear species can exist^23,49^. The exact nature of the latter has not been identified experimentally and cannot be inferred through comparison with classical macroscopic systems.

### Relativistic effects on the d(M) AOs

In a recent study^4^, performed at a higher level of relativistic theory than in ref. ^3^, Iliaš and Pershina concluded that the trend reversal in the FBDE from W to Sg is a result of the weakening of both the σ-forth and π-back donations. This is because of the relativistic energy destabilization of the 6d AOs in Sg. Generally, the (*n* − 1)d AOs expand when going down in group 6, see the maximum radial values, *R*_max_[(*n* − 1)d_5/2_/(*n* − 1)d_3/2_)] Mo(1.398/1.384 a.u.) < W(1.516/1.496 a.u.) < Sg(1.793/1.675 a.u.). However, the negative energies of those orbitals, *E*[(*n* − 1)d_5/2_/(*n* − 1)d_3/2_], increase from Mo to W and then decrease from W to Sg, that is, Mo(0.294/0.301 a.u) < W(0.324/0.348 a.u.) > Sg(0.257/0.297 a.u.)^50^. Because the group 6 M–CO bonding was shown to be of preferentially electrostatic nature^4^, the trend in the FBDE is defined exclusively by the trend in the AOs energies. The steady expansion of the (*n* − 1)d AOs of the metals, overlapping with molecular orbitals of the ligands, does not influence the trend in the FBDE.

## Online content

Any methods, additional references, Nature Portfolio reporting summaries, source data, extended data, supplementary information, acknowledgements, peer review information; details of author contributions and competing interests; and statements of data and code availability are available at https://doi.org/10.1038/s41586-026-11105-2.

## Supplementary information

Peer Review File

## Data Availability

The raw data are stored in the GSI data storage system. The data on observed ^259^Sg, ^255^Rf and ^161,162^W events are available at https://sf.gsi.de/library/c3889c02-9d88-44b5-b711-18b823edf624/Data%20Sg%20carbonyl%20chemistry%20experiment/, in compliance with data protection requirements for the use of the ‘GSI Seafile’. All data are password secured; please contact A.Y. or J. Khuyagbaatar.

## Code availability

The Monte Carlo code is based on the original algorithm published in ref. ^29^ and can be downloaded from https://sf.gsi.de/library/070e4c7a-4e42-4edb-96f8-8a57d5012731/Monte%20Carlo%20code%20for%20Sg(CO)6%20study/, in compliance with data protection requirements for the use of the ‘GSI Seafile’. All data are password secured; please contact A.Y.
