# Supplementary material for: Observation of relativistic bond weakening in seaborgium hexacarbonyl
Source: Nature. 2026 Oct 7;658(8135):337–41. doi: 10.1038/s41586-026-11105-2 (PMC13645635; doi:10.1038/s41586-026-11105-2)
Supplement: Supplementary file 1 [file 41586_2026_11105_MOESM1_ESM.pdf]

nature portfolio

Peer Review File

Observation of relativistic bond weakening in seaborgium hexacarbonyl

Corresponding Author: Dr Alexander Yakushev

Any redactions in this file are there to maintain patient confidentiality, the confidentiality of unpublished data, or to remove third-party material.

This file contains all reviewer reports in order by version, followed by all author rebuttals in order by version.

Attachments originally included by the reviewers as part of their assessment can be found at the end of this file.

Version 0:

Reviewer comments:

Referee #1

(Remarks to the Author)
The manuscript of Yakushev et al. details the assumed production of W(CO)6 and Sg(CO)6 molecules, wherein the relative formation of yields of the hexacarbonyl molecules, relative to all other species detected, is used to determine the first bond dissociation energy (FBDE) of the final CO for W versus Sg. The determination of the FBDE is performed using two sequential detectors, with the assumption that anything detected in the first detector is due to the decomposition of M(CO)6 and everything detected in the second detector is assumed to be only M(CO)6 molecules. The proportion of W(CO)6 and Sg(CO)6 observed relative to their respective decompositions then gives the relative FBDE of Sg(CO)6 versus W(CO)6.

The result here is that the bond dissociation energy of Sg(CO)6 is less than that of W(CO)6, which breaks the trend of expected periodic trend of increased CO binding down a group of metals. This break from expectations is attributed to the de-stabalization of the d-orbitals in Sg due to relativistic effects. This result is in agreement with theoretical predictions performed by a co-author of this work (V. Pershina), but are in disagreement with all previous theoretical work and the established experimental work in lighter transition metals that easier availability and spatial overlap for d-electrons to participate in back-bonding from the metal to the CO ligand strengthens the CO-metal bond. (See https://doi.org/10.1016/j.jms.2011.03.006)

This would be the first determination of a bond energy for a superheavy-element containing molecule, however the assumptions here about speciation and the relative chemical behavior of the different species believed to be present make the final conclusion difficult to justify so definitively as is stated in the manuscript. Without speciation of at least some component of the experiment, it is possible that this conclusion is misguided. I would not recommend publication of this result in Nature. However, as there are so few experimental results for superheavy element chemistry, this work should be published elsewhere, but with a less definitive conclusion. I offer some questions and considerations below.

On the need for speciation
In the introduction, Yakushev et al. discuss that previous work studying the decomposition of M(CO)6 complexes revealed that once equilibrium is reached, the fragmentation pattern following laser spectroscopy of the resulting products (confirmed with mass spec) is correlated to the individual bond energies of each sequential CO loss. This established chemistry is the foundation for the desire to measure the relative proportion of Sg(CO)6 detected on compact versus the decompositions (Sg(CO)6-n) on miniCompact. This is a valid foundation for an experiment if the speciation is clear. However, here the speciation of molecules exiting the recoil transfer chamber (RTC), being detected on miniCOMPACT, and being detected on COMPACT are assumed. Any incorrect assumptions here would invalidate the result.
Species produced in the RTC: From line 184, the assumption of the produced species that exit the RTC are unreacted atoms/ions, carbonyl complex fragments M(CO)6-n, and the fully coordinated M(CO)6. However, from the text it is not clear if the assumption is that the carbonyl complex fragments were once fully coordinated M(CO)6 that decomposed, or if they did not ever reach 6 carbonyls. Interestingly, this assumption appears to be different from the original referenced work of Evens et al. where the assumption was that only M(CO)6 was produced. A re-evaluation of the speciation of that work may also be

necessary given the considerations raised here. Also, why is it that the authors do not consider that M(CO)n ions are not produced? Similar gas phase work of metal ions with higher pressures (up to 3 atm) of CO show that cation complexes are produced as opposed to neutral molecules (see https://doi.org/10.1016/j.jms.2011.03.006). Below, I have included a figure from that reference showing the species produced when Mn+ is exposed to CO. The ionization energy of CO is 14 eV and the expected first ionization energy of Sg is predicted to be ~7.8 eV, it is very possible that Sg would not fully reduce and that cation complexes are more likely to be formed.

Species detected on miniCOMPACT/ COMPACT: Again from line 184, it is assumed that unreacted atoms/ions and carbonyl complex fragments M(CO)6-n are detected on miniCOMPACT. It is stated that these species are “non-volatile” and the expectation is that they will all stick to the SiO2 surface and that only M(CO)6 will be capable of moving on to the COMPACT detector. References are given to justify this assumption, however the experimental work of decomposition of assumed M(CO)6 on hot surfaces by Usoltsev et al. also does not include any speciation. Has there been any work demonstrating what surfaces and at what temperature carbonyl complex fragments adsorb that show a dramatic deviation from what the full carbonyl complex would adsorb too? Is it really infeasible that a M(CO)6-n complex could make it to COMPACT and perhaps also that a M(CO)6 species would be detected on miniCOMPACT? There must be some non-zero probability of each of these scenarios?

On the fractionation of species between COMPACT and miniCOMPACT:
In the referenced studies looking at the decomposition of carbonyl complexes with laser or mass spectroscopy, all observed species were from the decomposition of the carbonyl complex. As discussed above, it is not clear to me whether or not the authors of this work assumed that all species detected on miniCOMPACT are from the decomposition of the M(CO)6 complex or if they never achieved a full M(CO)6 complex. Also, it is not specified if they also assume decompositions could happen on the miniCOMPCT detector itself. The authors should clarify these points. If a complex never achieved a full M(CO)6 configuration, is the assumption here that the proportion of events detected on COMPACT versus miniCOMPACT is equivalent to the FBDE of the final CO addition still valid? Again see above figure where Mn+ species could be formed with less than six CO’s but at a higher pressure of CO than used here.
Also, if the fractionation pattern of lighter species produced by W versus Sg is different (for example Sg produced way more Sg(CO)5 than W produced W(CO)5) is just knowing the final production of M(CO)6 versus everything else still valid in leading to an extraction of the FBDE? Is it not necessary to know how much M(CO)6 versus M(CO)5 you have more specifically? What if other contamination molecules are present not correlated to the reactions with CO?

Referee #2

(Remarks to the Author)
Yakushev and coworkers report a thermodynamic study on the first bond dissociation energy (FBDE) of Sg(CO)6 and W(CO)6 with molecule at a time detection. This study provides a deeper understanding building off of the original synthesis of Sg(CO)6 from a few years back where they found that its volatility was similar to W(CO)6 and Mo(CO)6. It was found in this study, however, that there is a lower FBDE for Sg reversing the well-known trend that the hexacarbonyl complexes are more stable as one progresses down the Group VI transition metals. The explanation for this is that there is both weaker σ-donation from CO and weaker π-backdonation from Sg due to the indirect relativistic destabilization of 6d orbitals. Relativistic effects play a large role in chemical properties even in the 5d transition metals and the actinide series due to large spin-orbit and shielding effects. It is fascinating to see that these effects are so large in the transactinide series that they can completely change periodic trends. This study will provide the reader with an appreciation of these effects and how they can go against typical chemical intuition. Studying chemistry of elements this late in the periodic table is incredibly difficult, but fascinating and will likely also capture the general curiosity of the broad readership of Nature. The manuscript is well written, clear, and concise. I recommend publication following a few minor changes and enjoyed reading this paper.

Line 48: Consider adding the 6d10 electrons to Fl0 configuration
Line 63: Change “mass spectroscopy” to “mass spectrometry”
Change the hyphen to a minus sign in eqs. 1, 2 for the subscripts
In the sentence starting on Line 107 “The same authors…” consider stating both values for W(CO)6 and Sg(CO)6 and then state that the difference is 4 kJ/mol.
In line 124 please define “DC” in “DC-DFT”
When listing trends throughout the manuscript especially in line 127 it may be best to use consistent language or maybe simply state “Mo<W<Sg”
Line 139 change “…, along which a …” to “…, along with a…”
Line 154 “in-situ” is not in italics here but is elsewhere
Lines 226 and elsewhere there is a number with sub/superscript errors is it possible to change the hyphen to a minus sign?
Line 317 Change “stronger weakening” to something like “significant weakening”
Consider adding the number of Sg(CO)6 decay events to the abstract and conclusion.

Even though there is a flow rate given in the experimental section, it may be useful to give time frames on how long it would take the Sg(CO)6 to get to the COMPACT detector array.

My only suggestion for the manuscript overall is to add more discussion on relativistic effects. For the general audience, it may be helpful to add a qualitative description of why indirect relativistic effects result in the destabilization of the 6d orbitals. Furthermore, one could potentially make a counterargument due to the indirect relativistic expansion of the 6d orbitals and the greater ability to have orbital overlap and thus higher covalency. This is, to an extent, why 5f orbitals experience greater hybridization with ligand orbitals compared to 4f orbitals. I agree with the authors, however, that there is indeed a weakening of the bond because of the destabilization of the 6d orbitals and this destabilization effect is larger than the degree of 6d expansion since the 6d orbitals are already expanded beyond the radon core enough without the inclusion of relativistic effects to participate in covalent bonding. It may be useful to include some additional discussion here.

I congratulate the authors on these results and thank the authors and the editor for their time.

Referee #3

(Remarks to the Author)
A. Summary of the key results:

In their experiment Yakushev and co-authors employed the gas-filled magnetic separator TASCA (TransActinide Separator and Chemistry Apparatus) to create a beam of ^{259}Sg nuclei employing the nuclear reaction ^{208}Pb(^{52}Cr,1n)^{259}Sg. The nuclide ^{259}Sg decays by alpha-particle emission to the daughter nucleus ^{255}Rf with a half-life of ^{259g}Sg ($T_{1/2}$ = 402(56) ms). Furthermore, an isomeric state is known, which also decays by alpha-particle emission with a half-life of ^{259m}Sg ($T_{1/2}$ = 226(27) ms). The daughter nucleus ^{255}Rf is known to decay either by spontaneous fission or by alpha-particle emission to ^{251}No, which in turn decays by alpha-particle emission to ^{247}Fm. Therefore, ^{259}Sg can be unambiguously identified by registering decay-chains of the type α-sf or α-α-α with the correct alpha-decay energies and suitable decay times.

To investigate the lighter homolog element of group 6, W, the nuclides ^{161}W and ^{162}W were produced in the reaction ^{112}Sn(^{52}Cr,2-3n)^{161,162}W.

Behind the separator, the ^{259}Sg nuclei are stopped in a gas volume flushed with a 1:1 mixture of He and CO gas, in a so-called recoil transfer chamber. One can safely assume, that all ^{259}Sg nuclei are neutralized in this gas mixture and that their residual energy is sufficient to react with the CO molecules of the gas to form ^{259}Sg$(CO)_6$ or Sg-carbonyls with lower coordination number. These are multi-step decomposition and recombination processes, which result in a variety of metal–carbonyl species in a dynamic equilibrium.
Volatile and non-volatile species are rapidly transported by the gas flow to the miniCOMPACT detector, a channel formed by 32 pairs of PIN diodes with SiO^2 surfaces kept at room temperature that register alpha-particle- and spontaneous fission decays. Volatile species will most likely travel through the miniCOMPACT array without leaving a signal, while non-volatile species (non-volatile metal atoms and carbonyl fragments $M(CO)_n$ (1 ≥ n ≥ 5; M = ^{161,162}W, ^{259}Sg), deposit and form a distinct adsorption pattern indicative of a diffusion controlled deposition process.

Volatile species such as the hexacarbonyls of W and Sg will travel through miniCOMPACT and be registered in the COMPACT detector. Here the surfaces of the PIN diodes are covered with a thin layer of Au and a negative longitudinal temperature gradient is applied. Volatile species will deposit at a characteristic temperature and form a deposition peak.

The ratio of detected events in the COMPACT- and miniCOMPACT detector is indicative of the chemical interaction between $M(CO)_n$ (1 ≥ n ≥ 6; M = W, Sg) molecules with the detector surfaces at room temperature and the stability of $M(CO)_6$ molecules towards decomposition, i.e. the loss of one or more CO ligands. However, in the COMPACT detector almost exclusively only hexacarbonyl species are detected. With the known binding energy of CO in $W(CO)_6$ and from the ratio of count rates in the COMPACT- and miniCOMPACT detectors, the binding energy of CO in $Sg(CO)_6$ can be derived. Using a Monte-Carlo model simulating the transit of a hexacarbonyl species from the exit of the recoil transfer chamber to the deposition in COMPACT, the adsorption enthalpy on a Au surface can be determined as well.

The lower first bond dissociation energy (FBDE) in $Sg(CO)_6$ compared to $W(CO)_6$ has been predicted by state-of-the-art theoretical calculations, which predict a decreasing trend in bond strength from $W(CO)_6$ to $Sg(CO)_6$ due to the relativistic destabilization of the 6d atomic orbitals in Sg.

B. Originality and significance:

The experimental set-up is innovative and the combination of two detectors, an isothermal section and a gradient section indeed allows a relative evaluation of first bond dissociation energies of $W(CO)_6$ and $Sg(CO)_6$.

C. Data & methodology:

The critical points raised in the ensuing discussion will most likely not change the main outcomes of the submitted paper. However, in view of a thorough scientific discussion there are few points that need to be addressed.

A crucial quantity is the ratio of volatile to non-volatile fractions, i.e. the ratio of count rates in the miniCOMPACT and the COMPACT detector. This ratio depends, strictly speaking, on several variables: the first bond dissociation energy (FBDE), the gas low rate, the temperature, the nuclear half-live of the investigated species, and the volatility of the investigated species.

Furthermore, the authors assume, that in the miniCOMPACT only non-volatile species i.e. $M(CO)_n$ species with M = W, Sg and n = 0…5 are detected, while in COMPACT only $M(CO)_6$ is detected. This is not entirely correct. It is conceivable, that $M(CO)_6$ species are also registered in the miniCOMPACT, since this species can a) decay radioactively in flight or b) decay radioactively while adsorbed on the surface. Also volatile species migrate slower through an open column than the carrier gas as their interaction with the surface is significantly stronger than for an atom of the carrier gas. The inner volume of the miniCOMPACT detector is 0.96 cm^3, so the carrier gas at a flow rate of 2.5 L/min passes through the miniCOMPACT in 23 ms on average, which is relatively short compared to the half-lives of $^{259g,m}Sg$ of 402 and 226 ms. The connecting capillary between miniCOMPACT and COMPACT has a similar volume of 0.94 cm^3. By using the Monte Carlo model and the previously determined adsorption enthalpy of $M(CO)_6$ (M = W, Sg) on SiO_2-surface the fraction of $M(CO)_6$ detected in the miniCOMPACT could be calculated and shown to be negligible, but this discussion is missing.

The issue of different half-lives of ^{259g}Sg and ^{259m}Sg and ^{161}W and ^{162}W needs to be addressed. Actually, the half-lives of ^{161}W and ^{162}W have not been mentioned at all. These are $T_{1/2}(^{161}W)$ = 409(18) ms and $T_{1/2}(^{162}W)$ = 1.19(12) s. The half-life of the nuclide is an important parameter in the Monte Carlo modeling of the migration of a $M(CO)_6$ molecule through miniCOMPACT and COMPACT. The authors did not mention what the production ratio of $^{161}W/^{162}W$ is, or what half-life they used in the Monte Carlo model. The half-lives differ by almost a factor of 3. The same applies to ^{259g}Sg to ^{259m}Sg, which according to Ref. 39 are both produced simultaneously and the half-lives differ by a factor of about 2. Actually, the authors were not able to assign whether the decays originated from ^{259g}Sg or ^{259m}Sg. The influence of different half-lives in determining the FBDE and also the adsorption enthalpies in the Monte Carlo modeling are small and probably negligible, but the authors should discuss this fact and provide arguments why this can be neglected.

From previous studies it is known that the adsorption enthalpies of group 6 hexacarbonyls on SiO_2 surface are very similar. It seems therefore justified to simply determine the ratio of volatile and non-volatile fractions, but it could be mentioned in one or two sentences, why this is the case.

It appears from the presented data, that there were some minor issues in the operation of the miniCOMPACT and the COMPACT detector in experiments with $^{161,162}W$. It appears as if detector pair 15 in miniCOMPACT was not working, also it seems like only one side of detector pair 23 was operational in COMPACT.

D. Appropriate use of statistics and treatment of uncertainties

From the manuscript it appears as if 1 sigma uncertaninties are used. This is especially important to mention when discussing the 3 events of Sg in the COMPACT detector.

E. Conclusions: robustness, validity, reliability

In summary, the paper by Yakushev et al., demonstrates that on a one-atom-at-a-time level chemical experiments can be performed that allow to deduce the first bond dissociation energy of a molecule such as $Sg(CO)_6$ and that relativistic effects weaken the bond strength compared to the lighter homolog $W(CO)_6$.

F. Suggested improvements:

I recommend publication of the paper after the authors took care of the few points of criticism raised under C and D.

G. References:

All relevant references were cited.

H. Clarity and context:

The paper is well written and the provided graphs and drawings are of very good quality.

signed Prof. em. Dr. Andreas Türler

Version 1:

Reviewer comments:

Referee #1

(Remarks to the Author)
see attachment

Referee #3

(Remarks to the Author)
Comments to authors concerning the scientifc points raised in my referee report:

The authors have answered all my points of critism satisfactorily and revised the manuscript accordingly.

Concerning the discussion of speciation of the separated carbonyl compounds:

From a fundamental scientific point of view, the authors were unable to determine the speciation of the separated superheavy element compound. However, from the practical point of view this is currently not possible. In my view the authors have presented the relevant arguments that support the most likely interpretation to the best of our current understanding. It is permissible to draw conclusions from the current experiment, when all relevant arguments support the most likely interpretation of the experiment. One has to be careful not to compare apples with oranges. The synthesis and separation takes place in a real world environment, not in vacuum, and the investigated molecules are likely to be neutral species. Coordinatively unsaturated carbonyl species of group 6 elements have not been isolated to my knowledge in macroscopic quantities under the conditions of the experiment. It is conceivable that even single molecules of coordinatively unsaturated group 6 carbonyl species are less volatile than the hexacarbonyl and the probability for such a species to travel to the COMPACT detector without interaction with the walls of the RTC, the miniCOMPACT and the connecting capillary is very small.

The fact that the authors were unable to determine the speciation of Sg deposited in the COMPACT detector is due to the fact that state of the art technology is far from accomplishing such a task. The authors have performed an exceptionally difficult experiment and are reporting the result according to the highest scientific standards. I therefore remain with my recommendation that the paper should be accepted for publication by NATURE.

signed Prof.em. Dr. Andreas Türler

Open Access This Peer Review File is licensed under a Creative Commons Attribution 4.0 International License, which permits use, sharing, adaptation, distribution and reproduction in any medium or format, as long as you give appropriate credit to the original author(s) and the source, provide a link to the Creative Commons license, and indicate if changes were made.
In cases where reviewers are anonymous, credit should be given to 'Anonymous Referee' and the source.
The images or other third party material in this Peer Review File are included in the article's Creative Commons license, unless indicated otherwise in a credit line to the material. If material is not included in the article's Creative Commons license and your intended use is not permitted by statutory regulation or exceeds the permitted use, you will need to obtain permission directly from the copyright holder.

To view a copy of this license, visit https://creativecommons.org/licenses/by/4.0/

I appreciate the thoughtful responses to my comments and questions from the first round of review. In reading the comments of the other referees and responses from the authors of the manuscript, I am not yet convinced that a quantitative binding energy can be reported from a measurement that, as only plausibility arguments are made, is more qualitative in nature.

I have further comments and questions below.

On speciation:
In addressing my concerns regarding chemical speciation, the authors argue that the chemical community has not historically required direct speciation, citing past studies on hassium tetroxide and seaborgium hexacarbonyl. In the case of seaborgium hexacarbonyl, the chemical state was necessarily assumed and only indirectly verified by comparing its adsorption enthalpy on a quartz surface with theoretical predictions. However, more than 20 years after the initial chemical investigations of hassium, we must question whether the field should still accept inferred speciation as the standard, particularly as experiments become increasingly complex and a wider variety of assumed species are involved. Even if direct speciation is impossible for superheavy elements, modern techniques like time-of-flight mass spectrometry can identify the species produced for their higher-yield lighter homologues. If such direct speciation had been performed for the molecular tungsten species present in this study, the extrapolated assumptions regarding the chemical state of seaborgium would be significantly more convincing.

On extraction of the BDE:
For simplicity, let's assume all of the assumptions the manuscript makes on speciation is correct. Here, the author's argue that they can extract the BDE by simply comparing the amount of fully-coordinated $W(CO)_6$ and $Sg(CO)_6$ detected on compact to everything else detected on mini-compact. They take the ratio of these two detectors and then calculate the BDE for the Sg complex using equation 4, given that the BDE for the W complex has already been measured to be 192(8) kJ/mol.

The authors point to reference [19], stating that the "individual CO bond dissociation energies can be determined from a measurement of the individual fragment concentrations". However, in reading reference [19], it is stated in the conclusions that "It is premature to claim that individual CO bond dissociation energies can be determined unambiguously from these measurements".

I decided to test this by comparing the decomposition mass spectra for both $W(CO)_6$ and its lighter homologue $Mo(CO)_6$. The authors of this manuscript report the mass spectrum of $W(CO)_6$ in figure 4 as "the abundances of individual fragments in the equilibrated sate reflect their thermochemical and kinetic behavior (line 309)". Below I give the same mass spectrum of $W(CO)_6$ and also the mass spectrum of $Mo(CO)_6$ from NIST.

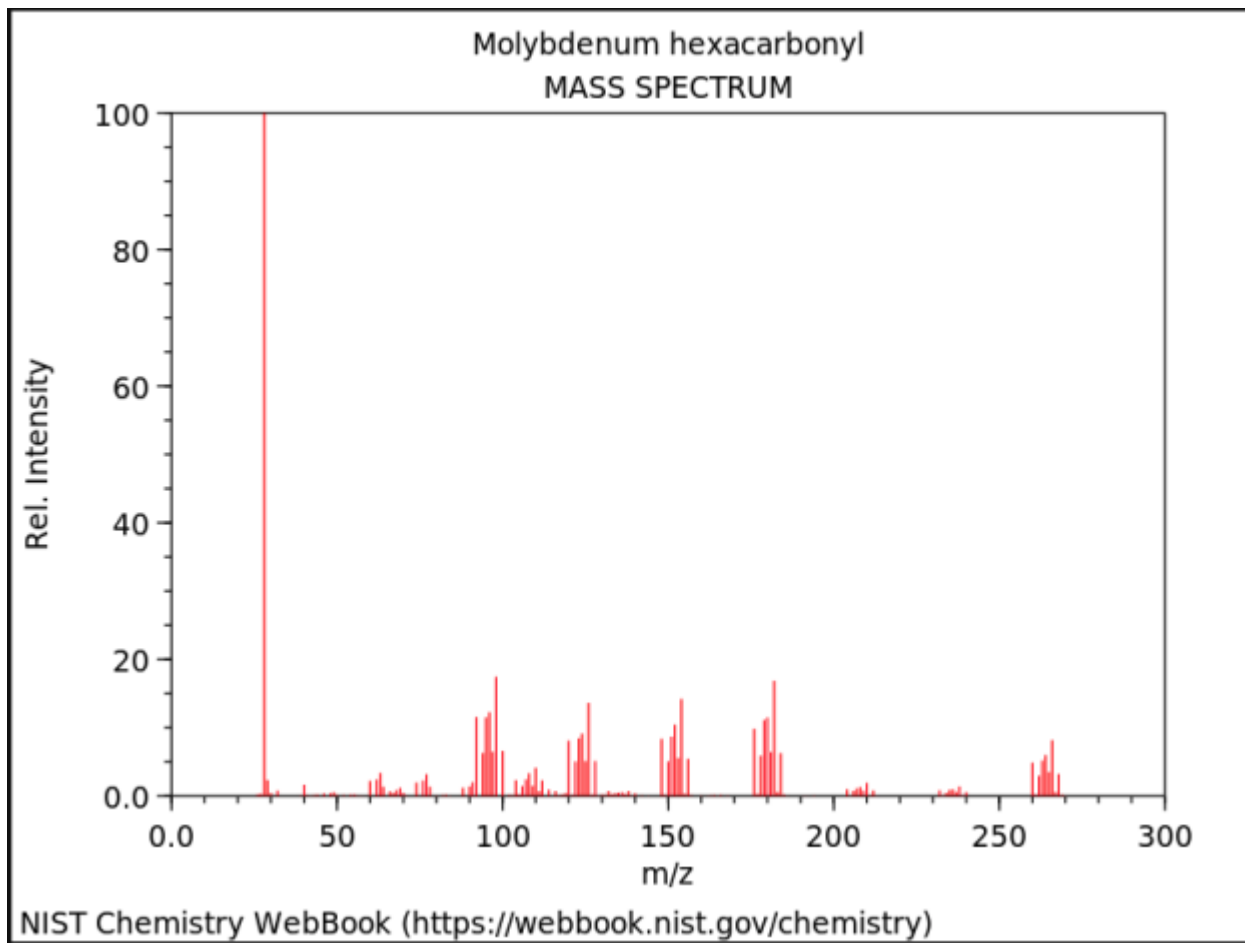

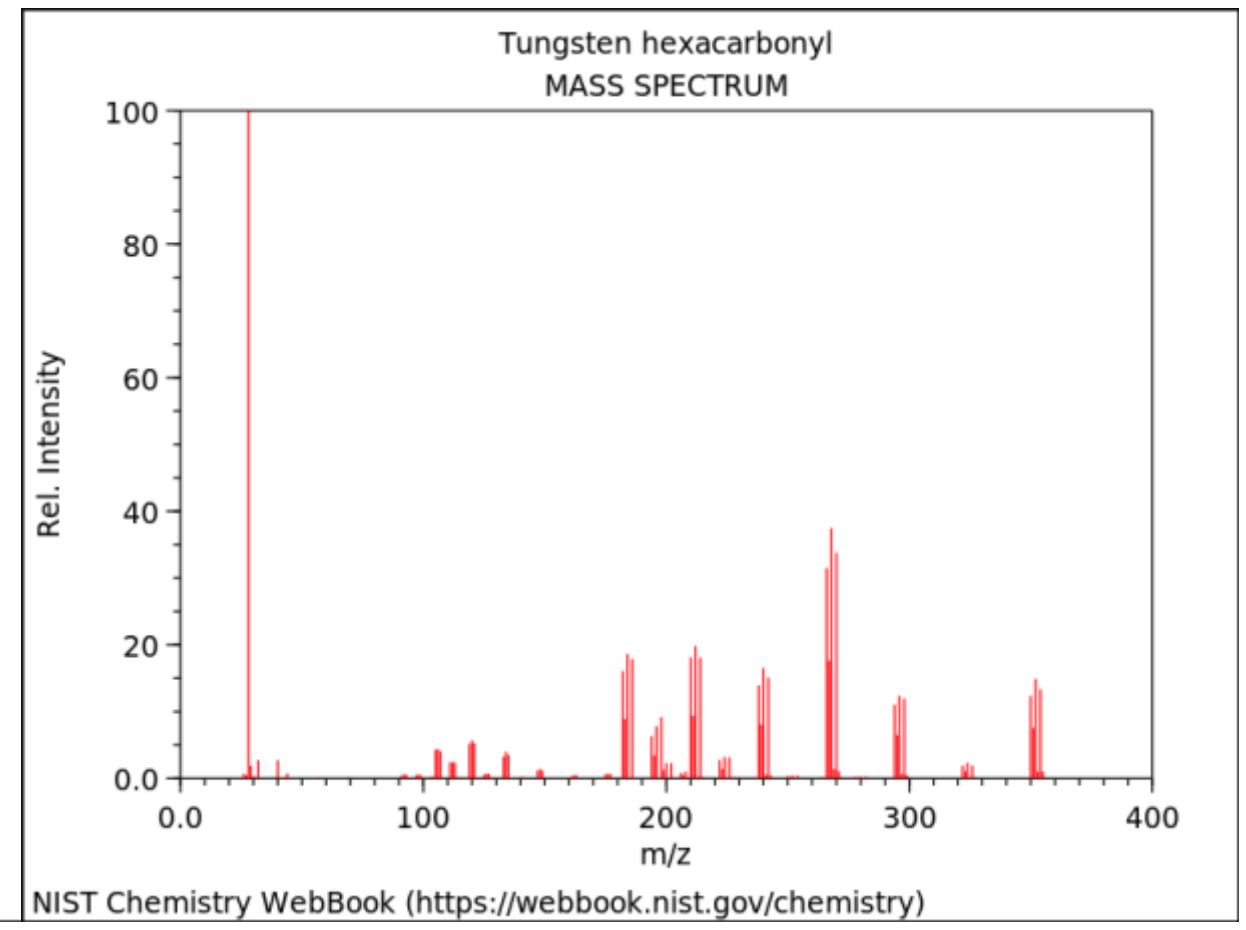

I compared the intensities from these spectra for the hexacarbonyl's versus the fragments and performed the same calculation from equation 4. My extracted intensities from these spectra are given in the table below. The BDE's for the sixth carbonyl of both of these species has been established, 192 for W and 169.45 for Mo. These are very different values, more different than what is being reported here for W and Sg. However, when I do this simple calculation, the ratio of $(CO)_6$ to $(CO)_{6-n}$ for both is effectively the same, ~0.12. What am I missing here? Can the authors use these data to validate the method they claim?

Species	Mo	W
M	18	18
M(CO)	14	20
$M(CO)_2$	14	17
$M(CO)_3$	15	38
$M(CO)_4$	2	14
$M(CO)_5$	2	5
$M(CO)_6$	8	15

$Mo(CO)_6 / Mo(CO)_{6-n} = 8/65 = 0.12$

$W(CO)_6 / W(CO)_{6-n} = 15/112 = 0.13$

The authors also confirm that it is expected for contaminant species and charged species to also be detected on the mini-compact detector. How does counting these species effectively as $(CO)_{6-n}$ species for the extraction of the FBDE not cause any issues?

On the final reported FBDE:

The authors comment that this result confirms “the trend of increasing FBDE values of hexacarbonyls in the group 6 is reversed from W to Sg (line 20)” and that this is “due to the destabilization of the 6d atomic orbitals in Sg (line 23)”. However, the reported value of 188(9) for Sg is effectively the same value as the 192(8) for W. Given the error bars here, can such a definitive statement be made?

Answers to the Referees

Referee #1 (Remarks to the Author):

The manuscript of Yakushev et al. details the assumed production of W(CO)6 and Sg(CO)6 molecules, wherein the relative formation of yields of the hexacarbonyl molecules, relative to all other species detected, is used to determine the first bond dissociation energy (FBDE) of the final CO for W versus Sg. The determination of the FBDE is performed using two sequential detectors, with the assumption that anything detected in the first detector is due to the decomposition of M(CO)6 and everything detected in the second detector is assumed to be only M(CO)6 molecules. The proportion of W(CO)6 and Sg(CO)6 observed relative to their respective decompositions then gives the relative FBDE of Sg(CO)6 versus W(CO)6.

The result here is that the bond dissociation energy of Sg(CO)6 is less than that of W(CO)6, which breaks the trend of expected periodic trend of increased CO binding down a group of metals. This break from expectations is attributed to the de-stabalization of the d-orbitals in Sg due to relativistic effects. This result is in agreement with theoretical predictions performed by a co-author of this work (V. Pershina), but are in disagreement with all previous theoretical work and the established experimental work in lighter transition metals that easier availability and spatial overlap for d-electrons to participate in back-bonding from the metal to the CO ligand strengthens the CO-metal bond. (See https://doi.org/10.1016/j.jms.2011.03.006)

Dear Referee,

We thank the referee for the strong interest in our work and for the detailed analysis. Indeed, the weakening of the binding energy in the Sg(CO)6 molecule predicted almost ten years ago (Ilias&Pershina, ref. 28 in the original submission) disagrees with the older work performed in 1999 by Nash&Bursten. The calculations by Ilias and Pershina were performed using theoretical methods at a significantly higher level of modern relativistic theory, e.g., including the scalar-relativistic effect and spin-orbital splitting. The results of these calculations reproduce very well the experimental values for the lighter homologs, and, thus, also the increasing trend from Cr to W. On this basis, they predict a lower FBDE value for Sg as compared to W, clearly showing the trend reversal caused by the increasing relativistic effects in Sg. Nash&Bursten used a method with some drawbacks (s. Discussion section of our manuscript), and could not reproduce the known experimental values of the homologs. Dr. Pershina, a well-acknowledged theoretician in the field of superheavy element (SHE) chemistry, is a member of the Superheavy Element Chemistry group at GSI, and thus most naturally, she was involved in the present study.

This would be the first determination of a bond energy for a superheavy-element containing molecule, however the assumptions here about speciation and the relative chemical behavior of the different species believed to be present make the final conclusion difficult to justify so definitively as is stated in the manuscript. Without speciation of at least some component of the experiment, it is possible that this conclusion is misguided. I would not recommend publication of this result in Nature. However, as there are so few experimental results for superheavy element chemistry, this work should be published elsewhere, but with a less definitive conclusion. I offer some questions and considerations below.

Answer:

We agree with the referee that definite speciation of SHE containing compounds would be ideal. To date, though, that has never been achieved because of the limitations inherent in studies of such chemical systems. The unambiguous speciation of SHE compounds by classical methods, e.g., infrared spectroscopy, is not possible due to the single atom or molecule at-a-time situation in SHE chemistry experiments. We understand that at first glance, a lack of direct speciation may be seen as a fundamental problem. The experimental chemical knowledge in this part of the periodic table was forced to rely on well-known and proven established trends in the periodic table and on theoretical work to arrive at the current state of knowledge. Thus, in all cases involving SHE-containing compounds the assignment to a specific compound (speciation) is always made based on the known properties of homologs and on the theoretical predictions.

One of the prime examples, carried out by a collaboration where several co-authors of the present study, as well as authors from other international laboratories, were involved, concerns the characterization of hassium as the tetroxide. Being a member of group 8 and a homolog of osmium, the observation of a volatile oxide of hassium (Düllmann, Ch. E. et al. Chemical investigation of hassium (element 108). *Nature* **418**, 859–862 (2002) https://doi.org/10.1038/480 nature00980) was accepted by the chemical community as a proof for the speciation as the tetroxide. No other oxidic species exhibit such an extraordinarily high volatility in this group, but volatile tetroxides are known for both Ru and Os. Further examples from this field exist:

i) Cn was studied in a pure He-Ar atmosphere and characterized as a volatile metal, more inert than its nearest homolog Hg (Eichler, R. et al. Chemical characterization of element 112. *Nature* 447, 72–75 (2007) https://doi.org/10.1038/nature05761).
ii) From the results of the first synthesis of the Sg(CO)6 complex and from the adsorption studies of M(CO)6 (M = Mo, W, Sg) it was concluded that the volatile and inert hexacarbonyl complex of Sg was formed and studied (Even, J. et al. Synthesis and detection of a seaborgium carbonyl complex. *Science* **345**, 1491–1493 (2014) https://doi.org/10.1126/science.1255720).

The hexacarbonyl complexes are well-studied compounds. They are volatile due to their octahedral symmetry and zero dipole moment. They show a high volatility in the gas phase and a low interaction to solid surfaces upon adsorption. These well-known chemical properties were extended to Sg(CO)6 in the work by Even et al. and allowed us undoubtedly to assign the chemical compound of Sg detected at a low temperature in COMPACT to Sg(CO)6. Besides hexacarbonyl complexes of group 6 metals, only halides in their highest oxidation state exhibit high volatility. The formation of such halides can be ruled out under our experimental conditions.

In principle, a powerful **complementary** way to support the chemical speciation is the mass measurement. Recently, this method was applied for the identification of doubly charged molecular ions – the complex cations of Ac and No with neutral ligands, H2O and N2 (Ref 41 in the original manuscript, Pore, J. L. et al. Direct identification of Ac and No molecules with an atom-at-a-time technique. *Nature* **644**, 376–380 (2025) https://doi.org/10.1038/s41586-025-09342-y). Moderate mass resolution and the knowledge of the building blocks of the compounds allowed for the assignment of the detected chemical compounds. However, still the chemical speciation was not performed directly and it is also based on several assumptions. Note, that the mass measurement is based on the extraction, mass selection and identification of cationic species, separated in vacuum by means of electrical and magnetic fields. This is an environment that differs substantially from that employed in our work. Furthermore, the application of this method for SHE compounds is currently unfeasible due to sensitivity limitations (the method was proved for single ions of

Ac and No produced with cross sections, which are larger than production cross sections of Sg isotopes by a factor of more than 10^3.

And finally, due to the equal mass of the CO and N2 molecules, even a mass identification will not provide the unambiguous chemical formula for the case of carbonyl complexes M(CO)x.

On the need for speciation

In the introduction, Yakushev et al. discuss that previous work studying the decomposition of M(CO)6 complexes revealed that once equilibrium is reached, the fragmentation pattern following laser spectroscopy of the resulting products (confirmed with mass spec) is correlated to the individual bond energies of each sequential CO loss. This established chemistry is the foundation for the desire to measure the relative proportion of Sg(CO)6 detected on compact versus the decompositions (Sg(CO)6-n) on miniCompact. This is a valid foundation for an experiment if the speciation is clear. However, here the speciation of molecules exiting the recoil transfer chamber (RTC), being detected on miniCOMPACT, and being detected on COMPACT are assumed. Any incorrect assumptions here would invalidate the result.

Answer:

It was not our intention to study the decomposition of M(CO)6. In macro chemistry, studies of non-saturated carbonyl complexes are performed only by decomposition of the hexacarbonyl complex because non-saturated fragments are not available as stable compounds. We studied the formation of the hexacarbonyl complex starting from the bare metal ion/atom in a multi-step reversible process. The carbonyl species are separated in the gas-chromatography channel in accordance with their reactivity and volatility. The assumption that species detected in the second detector are hexacarbonyl complexes W(CO)6 and Sg(CO)6 is based on their known properties (volatility, enthalpy of adsorption, see above). All other non-saturated carbonyl complex fragments do not fulfil the 18-electron rule, have structures with lower symmetries, and, thus, higher dipole moments, and are significantly more reactive than the hexacarbonyl complex. Studies of the thermal decomposition of hexacarbonyl complexes on different surfaces have demonstrated that upon elimination of one CO ligand the residual molecule readily adsorbs on a surface, even at elevated temperatures (Usoltsev, I. et al. Decomposition studies of group 6 hexacarbonyl complexes. Part 1: Production and decomposition of $Mo(CO)_6$ and $W(CO)_6$. *Radiochim. Acta* **104**, 141-151 (2016) https://doi.org/10.1515/ract-2015-2445).

Species produced in the RTC: From line 184, the assumption of the produced species that exit the RTC are unreacted atoms/ions, carbonyl complex fragments M(CO)6-n, and the fully coordinated M(CO)6. However, from the text it is not clear if the assumption is that the carbonyl complex fragments were once fully coordinated M(CO)6 that decomposed, or if they did not ever reach 6 carbonyls.

Answer:

A new sentence is added to clarify the text:

"All products of the multi-step process of the carbonyl complex formation and decomposition, which occurs inside the RTC, are extracted as cations or neutrals by the gas flow into the first detection channel, miniCOMPACT, except for those that adsorbed on the surface inside the RTC owing to finite extraction yield."

Interestingly, this assumption appears to be different from the original referenced work of Evens et al. where the assumption was that only M(CO)6 was produced.

Answer:

We disagree with this comment. In the original work of Even et al. the aim of study was the formation of the volatile hexacarbonyl complex of Sg, similarly to the lighter homologs, Mo and W. The experimental setup used in that work was sensitive only to volatile compounds. Note that a rather low yield (a few percent) was observed in the first and in the following studies (Eichler, R. et al. Complex chemistry with complex compounds. *EPJ Web of Conferences* **131**, 07005 (2016) https://doi.org/10.1051/epjconf/201613107005). The missing yield (>90%) was likely species that did not reach COMPACT in that experiment and remained unobserved. In the present experiment, miniCOMPACT was added to register also species other than M(CO)6.

A re-evaluation of the speciation of that work may also be necessary given the considerations raised here. Also, why is it that the authors do not consider that M(CO)n ions are not produced?

Answer:

We have answered to this comment above, cf. the inserted sentence "All products of the multi-step process….".

Similar gas phase work of metal ions with higher pressures (up to 3 atm) of CO show that cation complexes are produced as opposed to neutral molecules (see https://doi.org/10.1016/j.jms.2011.03.006). Below, I have included a figure from that reference showing the species produced when Mn+ is exposed to CO. The ionization energy of CO is 14 eV and the expected first ionization energy of Sg is predicted to be ~7.8 eV, it is very possible that Sg would not fully reduce and that cation complexes are more likely to be formed.

Answer:

The paper mentioned by the referee describes the reaction between metal ions produced by laser ablation in a vacuum environment with the expanding gas from a pressurized volume (3-10 bars) into vacuum. Metal complexes, which are formed in the plasma and cooled by the expanding gas, stay in cationic form and can be selected by mass, e.g., by a time-of-flight spectrometer. Within such instruments, operated under high vacuum conditions, these ions are guided by electrical und magnetic fields without contact with the surface. Again, this is an environment that differs substantially from that employed in our work.

Species detected on miniCOMPACT/ COMPACT: Again from line 184, it is assumed that unreacted atoms/ions and carbonyl complex fragments M(CO)6-n are detected on miniCOMPACT. It is stated that these species are "non-volatile" and the expectation is that they will all stick to the SiO2 surface and that only M(CO)6 will be capable of moving on to the COMPACT detector. References are given to justify this assumption, however the experimental work of decomposition of assumed M(CO)6 on hot surfaces by Usoltsev et al. also does not include any speciation. Has there been any work demonstrating what surfaces and at what temperature carbonyl complex fragments adsorb that show a dramatic deviation from what the full carbonyl complex would adsorb too? Is it really infeasible that a M(CO)6-n complex could make it to COMPACT and perhaps also that a M(CO)6 species would be detected on miniCOMPACT? There must be some non-zero probability of each of these scenarios?

Answer:

The answer is given by the long history of the production and separation of the saturated carbonyl complexes, which fulfil the 18-electrons rule. In 1890, Ludwig Mond discovered a process that allowed for purification of Fe and Ni using the high volatility of carbonyl complexes Fe(CO)5 and Ni(CO)4. They can be produced from the elements (metal powder) and CO gas at 50-75 °C and isolated by distillation. Further, they can be decomposed at elevated temperatures (175-250 °C). For the group 6, such a process is thermodynamically possible, but it needs very high CO pressures. Thus, the reductive carbonylation is applied for the W(CO)6 production. Only the volatile carbonyl complex M(CO)6 is known for group 6. Complexes with fewer CO ligands were identified only in decomposition studies of the hexacarbonyl complexes, e.g., by in-situ mass spectrometric or photometric studies. However, they could not be isolated as compounds (refs 13-21 of the original manuscript).

The thermal decomposition of hexacarbonyls is particularly important to grow thin films of refractory metals, such as W, in the CVD processes, by using "*a dramatic deviation*" in the volatility between the volatile precursor (W(CO)6) and the growing films, containing W, C and O atoms. The temperature of the hot surface, on which the decomposition occurs, depends on the kind of the surface. So, W(CO)6 decomposes on the W(110) surface at 200K, whereas W(CO)6-n species remain on the surface. The undecomposed W(CO)6 was desorbed by heating the surface to 200 K (-73 °C) in vacuum. (F.A. Flitsch, J.R. Swanson' and C.M. Friend. Thermal and photoinduced decomposition of W(CO)6 on W(110). *Surface Science* 245 (1991) 85-95). However, temperatures above 300°C are needed for the film creation by CVD of W from W(CO)6 on silicon structures (V. V. Krisyuk et al.: Thermal decomposition of tungsten hexacarbonyl. *Phys. Status Solidi C* **12**, No. 7 (2015)). The surface effect was also observed during decomposition studies in the work by Usoltsev et al.; above mentioned by the referee.

Thus, it is really infeasible that M(CO)6-n fragments could reach the second COMPACT. However, the in-flight nuclear decay of 259Sg(CO)6 in the first detector is possible - less than 1 out of 72 decay chains from 259Sg could be assigned to the decay in flight. We have added a numerical analysis of the probability of the decay of 259Sg during the transport of the Sg(CO)6 complex through the miniCOMPACT to the text:

"Based on Monte Carlo simulations (described in the following section), the probability for decay in flight of volatile $^{259m,g}Sg(CO)_6$ was estimated (~16%) to be less than one event out of 72 $^{259m,g}Sg$ events registered in miniCOMPACT. This would change the ratio of volatile and non-volatile fractions to 0.05, which lies within the statistical error bars of the value $0.04^{+0.05}_{-0.02}$."

On the fractionation of species between COMPACT and miniCOMPACT:
In the referenced studies looking at the decomposition of carbonyl complexes with laser or mass spectroscopy, all observed species were from the decomposition of the carbonyl complex. As discussed above, it is not clear to me whether or not the authors of this work assumed that all species detected on miniCOMPACT are from the decomposition of the M(CO)6 complex or if they never achieved a full M(CO)6 complex.

Answer

As we have mentioned above and in the original manuscript, the non-volatile species detected in miniCOMPACT are the reaction products of both formation and decomposition processes. Each individual step in the formation process is reversible and is accompanied by the

decomposition step, especially taking into account the presence of ions, electrons, neutrons and photons inside the RTC volume.

Also, it is not specified if they also assume decompositions could happen on the miniCOMPCT detector itself. The authors should clarify these points.

Answer:

For both W and Sg, the complex decomposition and recombination with CO can also happen inside the detector channels on the surface or in the gas phase.

If a complex never achieved a full M(CO)6 configuration, is the assumption here that the proportion of events detected on COMPACT versus miniCOMPACT is equivalent to the FBDE of the final CO addition still valid?

Answer:

Yes, it is. The value of FBDE is a measure of the reaction rate for the last addition or first decomposition step. A larger FBDE value means a larger rate constant for the M(CO)6 formation and a lower rate for the decomposition of M(CO)6. Thus, a larger FBDE value is responsible for a higher stability and a higher concentration in the reaction mixture. Because the Sg(CO)6 formation rate was measured relative to that of W(CO)6 under identical conditions, a larger fraction of W(CO)6 complex in the mixture corresponds to a larger FBDE value. Similarly, larger peaks from the fragments M(CO)x in mass spectra of M(CO)6 correspond to larger values of the bond dissociation energy, as was shown by many works on M(CO)6 decomposition cited in the manuscript.

Again see above figure where Mn+ species could be formed with less than six CO's but at a higher pressure of CO than used here.

Answer:

We did not find any figures in the report. Once again, the paper mentioned by the Referee describes the formation of carbonyl complexes with the **cooling expanding** CO gas in the vacuum environment, and thus, it is not relevant to our experiment. The method utilizes CO gas at stagnation pressures of 3–10 bar **inside a gas cylinder**. The CO gas is introduced into the reaction zone via a pulse valve expands into the low-pressure zone, and cools the metal ions vaporized by a laser. There is no reaction taking place in a 3-bar environment in that work.

Also, if the fractionation pattern of lighter species produced by W versus Sg is different (for example Sg produced way more Sg(CO)5 than W produced W(CO)5) is just knowing the final production of M(CO)6 versus everything else still valid in leading to an extraction of the FBDE? Is it not necessary to know how much M(CO)6 versus M(CO)5 you have more specifically?

Answer:

Since the formation energy of all hexacarbonyl complexes is nearly constant, the ratio of a hexacarbonyl fragment to the sum of all other fragments is a measure of the stability of the hexacarbonyl complex. Thus, it is not necessary to know the concentration of the M(CO)5 complex. If the formation of carbonyl complexes could be studied using, e.g., the FIONA technique described in the above-mentioned publication by J.L Pore et al., one can obtain the fraction of all individual carbonyl fragments for homologs (Mo and W) (the full mass spectrum) and compare to the known mass spectrum. The measured mass spectrum of all Sg carbonyl fragments will allow for the determination of bond dissociation energies of all

individual carbonyl fragments. This possible application is added to the outlook in the revised version:

“By application of the method of compound determination by the mass analysis to carbonyl complexes, all charged carbonyl fragments can be identified in future experiments on the single-atom level, resulting in the chemical speciation and determination of bond dissociation energies for individual fragments. This would be especially important for carbonyl complexes with odd-Z elements, where, unlike macroscopic amounts, only mononuclear species exist in the atom-at-a-time regime (Refs 31, 38, 39). The exact nature of latter has not been identified experimentally and cannot be inferred via comparison to classical microscopic systems.”

What if other contamination molecules are present not correlated to the reactions with CO?
Answer:

In the present experiment all common gas contaminants (H2O, O2) were kept at the one ppm level in the He-CO gas mixture (1:1) at one bar. Gas-phase chemical reactions involving gas contaminants cannot be excluded but are statistically much less probable than gas-phase reactions with CO molecules. Metal species, e.g., containing impurity ligands (H2O, O2, N2), have a lower volatility than M(CO)6 and would be also adsorbed in the first detector.

Referee #2

Yakushev and coworkers report a thermodynamic study on the first bond dissociation energy (FBDE) of Sg(CO)6 and W(CO)6 with molecule at a time detection. This study provides a deeper understanding building off of the original synthesis of Sg(CO)6 from a few years back where they found that its volatility was similar to W(CO)6 and Mo(CO)6. It was found in this study, however, that there is a lower FBDE for Sg reversing the well-known trend that the hexacarbonyl complexes are more stable as one progresses down the Group VI transition metals. The explanation for this is that there is both weaker σ-donation from CO and weaker π-backdonation from Sg due to the indirect relativistic destabilization of 6*d* orbitals. Relativistic effects play a large role in chemical properties even in the 5*d* transition metals and the actinide series due to large spin-orbit and shielding effects. It is fascinating to see that these effects are so large in the transactinide series that they can completely change periodic trends. This study will provide the reader with an appreciation of these effects and how they can go against typical chemical intuition. Studying chemistry of elements this late in the periodic table is incredibly difficult, but fascinating and will likely also capture the general curiosity of the broad readership of *Nature*. The manuscript is well written, clear, and concise. I recommend publication following a few minor changes and enjoyed reading this paper.

Dear Referee,
Thank you very much for your positive and constructive report. We are glad that you valued the main idea of our study, which we aimed to convey to the reader: revealing violation of the periodic trend within a PTE group caused by relativistic effects. We consider your suggestion to add some more theoretical considerations and explanations of the nature of the relativistic influence on bond strength in carbonyl complexes to be very valuable. In our experimental work, we primarily focused on the experiment, referring to support from previously published theoretical work (ref. 28 in original submission). Nevertheless, these suggestions undoubtedly helped improve our presentation. All your comments have been incorporated into the text of the revised manuscript and are presented below.

Line 48: Consider adding the 6*d*10 electrons to Fl0 configuration
Answer: We have added 6d10 electrons to the Fl configuration.
Line 63: Change “mass spectroscopy” to “mass spectrometry”
Answer: The change is made.
Change the hyphen to a minus sign in *eqs*. 1, 2 for the subscripts
Answer: The change is made.
In the sentence starting on Line 107 “The same authors…” consider stating both values for W(CO)6 and Sg(CO)6 and then state that the difference is 4 kJ/mol.
Answer:
Because the FBDE values for W(CO)6 and Sg(CO)6 are given in kcal/mol in work by Nash&Bursten, we wanted to avoid usage of two different energy units. However, we agree with the referee’s suggestion and rephrased this sentence in the revised version as follows:

"The same authors later calculated the FBDE of $W(CO)_6$ and $Sg(CO)_6$ using the Relativistic Effective Core Potentials (RECPs) and suggested that the experimental FBDE value should increase from 46(2) kcal /mol for $W(CO)_6$ to 47(2) kcal/mol for $Sg(CO)_6$."

In line 124 please define "DC" in "DC-DFT"

Answer:

Thank you for the comment. The Dirac-Coulomb methods were already mentioned in the previous sentence starting on Line 120, however without the abbreviation. We corrected this mistake. The sentence on Line 120 now includes the definition of DC (s. below) so that we do not need to define DC in the next sentence.

"The most recent FBDE-calculations for $Mo(CO)_6$, $W(CO)_6$ and $Sg(CO)_6$ were performed by Iliaš and Pershina using several nonrelativistic and relativistic DFT approaches, as well as *ab-initio* Dirac-Coulomb (DC) correlated methods, including Coupled Cluster Singles, Doubles and perturbative Triples (CCSD(T))."

When listing trends throughout the manuscript especially in line 127 it may be best to use consistent language or maybe simply state "Mo<W<Sg"

Answer: The sentence on Line 127 was changed to

"The non-relativistic values were, on the contrary, increasing from Mo to Sg, i.e., Mo<W<Sg."

Line 139 change "…, along which a …" to "…, along with a…"

Answer: The word "which" is correct here because; "a negative temperature gradient was applied" along "a SiO2-covered chromatography column". No changes were made.

Line 154 "in-situ" is not in italics here but is elsewhere

Answer: Thank you, "in-situ" was changed to italics through the whole manuscript.

Lines 226 and elsewhere there is a number with sub/superscript errors is it possible to change the hyphen to a minus sign?

Answer: We changed the hyphen before the number with subscript errors to the minus sign through the whole manuscript.

Line 317 Change "stronger weakening" to something like "significant weakening"

Answer: The change was made to "more substantial weakening".

Consider adding the number of Sg(CO)6 decay events to the abstract and conclusion.

Answer:

Thank you for the suggestion. Because not only the number of Sg(CO)6 events is important, but also the ratio between volatile and non-volatile fractions, we added one sentence to the Conclusion section:

"Three out of 75 registered events from ^{259}Sg were found in the second detector array and assigned to the $^{259}Sg(CO)_6$ complex."

Even though there is a flow rate given in the experimental section, it may be useful to give time frames on how long it would take the Sg(CO)6 to get to the COMPACT detector array.

Answer:

We have extended the sentence in the Methods section as follows:

"The gas mixture of 50% He (99.9999%) and 50% CO (99.997%) circulated in a closed loop at a flow rate of 2.5 l/min. Less than 100 ms are required to flush out the whole detection setup."

My only suggestion for the manuscript overall is to add more discussion on relativistic effects. For the general audience, it may be helpful to add a qualitative description of why indirect relativistic effects result in the destabilization of the 6*d* orbitals.

Answer:

Thanks. We added a sentence to the paragraph starting on Line 51 and an additional reference (Pyykkö, P. Relativistic Effects in Structural Chemistry. *Chem. Rev.* **88(3)**, 563-594 (1988) https://doi.org/10.1021/cr00085a006):

"The second indirect relativistic effect on the 6d orbitals is due to their screening from the nucleus by the relativistically contracted inner s and $p_{1/2}$ shells."

Furthermore, one could potentially make a counterargument due to the indirect relativistic expansion of the 6*d* orbitals and the greater ability to have orbital overlap and thus higher covalency. This is, to an extent, why 5*f* orbitals experience greater hybridization with ligand orbitals compared to 4*f* orbitals. I agree with the authors, however, that there is indeed a weakening of the bond because of the destabilization of the 6*d* orbitals and this destabilization effect is larger than the degree of 6*d* expansion since the 6*d* orbitals are already expanded beyond the radon core enough without the inclusion of relativistic effects to participate in covalent bonding. It may be useful to include some additional discussion here.

I congratulate the authors on these results and thank the authors and the editor for their time.

Answer:

Thank you for this comment. Even though this analysis, i.e., comparing trends in the covalent and ionic bonding, should have been given in the original theory paper (Ref. 28 in the original manuscript), we are happy to add this discussion here. This additional text on p. 6 reads as follows:

"It is interesting to note here that the trend in the expansion of the (n-1)d AOs in group 6 is to a steady increase, as their maximum radial values, $R_{max}[(n\text{-}1)d_{5/2}/(n\text{-}1)d_{3/2})]$, increase from Mo to Sg, i.e., Mo(1.398/1.384 a.u.) < W(1.516/1.496 a.u.) < Sg(1.793/1.675 a.u.), while their negative energies, $E_{max}[(n\text{-}1)d_{5/2}/(n\text{-}1)d_{3/2}]$, increase from Mo to W and then decrease from W to Sg, i.e., Mo(0.294/0.301 a.u) < W(0.324/0.348 a.u.) > Sg(0.257/0.297 a.u.). Since the M-(CO) bonding in the $M(CO)_6$ complexes was shown to be of preferentially electrostatic nature, the steady expansion of the (n-1)d AOs in the group, as overlapping with AOs of the ligands, does not influence the trend. The latter is defined exclusively by the shape of the AOs energy trend."

The reference on the DF values was added to this paragraph (J.P. Desclaux, Atomic Data Nucl. *Atomic Data and Nuclear Data Tables* **12**, 311-406 (1973)).

We have also made small corrections in the text, like adding missing data on the electronic ground state of Sg.

Referee #3 (Remarks to the Author):

Dear Prof. Andreas Türler,

We sincerely thank you for the high praise we received from you as a recognized expert in the chemistry of superheavy elements, who was actively involved in chemical studies of several superheavy elements. As a profound expert on the experiment, you were able to note a number of important details that were omitted from the original text, primarily due to the limited space. Many of your comments concern details that were taken into account already during the data analysis, but were not described fully in the text. Thanks to your comments, the text becomes now more clear and less ambiguous.

A. Summary of the key results:

In their experiment Yakushev and co-authors employed the gas-filled magnetic separator TASCA (TransActinide Separator and Chemistry Apparatus) to create a beam of ^{259}Sg nuclei employing the nuclear reaction $^{208}Pb(^{52}Cr,1n)^{259}Sg$. The nuclide ^{259}Sg decays by alpha-particle emission to the daughter nucleus ^{255}Rf with a half-life of ^{259g}Sg ($T_{1/2}$ = 402(56) ms). Furthermore, an isomeric state is known, which also decays by alpha-particle emission with a half-life of ^{259m}Sg ($T_{1/2}$ = 226(27) ms). The daughter nucleus ^{255}Rf is known to decay either by spontaneous fission or by alpha-particle emission to ^{251}No, which in turn decays by alpha-particle emission to ^{247}Fm. Therefore, ^{259}Sg can be unambiguously identified by registering decay-chains of the type α-sf or α-α-α with the correct alpha-decay energies and suitable decay times.

To investigate the lighter homolog element of group 6, W, the nuclides ^{161}W and ^{162}W were produced in the reaction $^{112}Sn(^{52}Cr,2\text{-}3n)^{161,162}W$.

Behind the separator, the ^{259}Sg nuclei are stopped in a gas volume flushed with a 1:1 mixture of He and CO gas, in a so-called recoil transfer chamber. One can safely assume, that all ^{259}Sg nuclei are neutralized in this gas mixture and that their residual energy is sufficient to react with the CO molecules of the gas to form $^{259}Sg(CO)_6$ or Sg-carbonyls with lower coordination number. These are multi-step decomposition and recombination processes, which result in a variety of metal–carbonyl species in a dynamic equilibrium.
Volatile and non-volatile species are rapidly transported by the gas flow to the miniCOMPACT detector, a channel formed by 32 pairs of PIN diodes with SiO^2 surfaces kept at room temperature that register alpha-particle- and spontaneous fission decays. Volatile species will most likely travel through the miniCOMPACT array without leaving a signal, while non-volatile species (non-volatile metal atoms and carbonyl fragments $M(CO)_n$ ($1 \geq n \geq 5$; M = $^{161,162}W$, ^{259}Sg), deposit and form a distinct adsorption pattern indicative of a diffusion controlled deposition process.

Volatile species such as the hexacarbonyls of W and Sg will travel through miniCOMPACT and be registered in the COMPACT detector. Here the surfaces of the PIN diodes are covered with a thin layer of Au and a negative longitudinal temperature gradient is applied. Volatile species will deposit at a characteristic temperature and form a deposition peak.

The ratio of detected events in the COMPACT- and miniCOMPACT detector is indicative of the chemical interaction between $M(CO)_n$ ($1 \geq n \geq 6$; M = W, Sg) molecules with the detector surfaces at room temperature and the stability of $M(CO)_6$ molecules towards decomposition, i.e. the loss of one or more CO ligands. However, in the COMPACT detector almost exclusively only hexacarbonyl species are detected. With the known binding energy of CO in $W(CO)_6$ and from the ratio of count rates in the COMPACT- and miniCOMPACT detectors, the binding energy of CO in $Sg(CO)_6$ can be derived. Using a Monte-Carlo model simulating the transit of a hexacarbonyl species from the exit of the recoil transfer chamber to the deposition in COMPACT, the adsorption enthalpy on a Au surface can be determined as well.

The lower first bond dissociation energy (FBDE) in $Sg(CO)_6$ compared to $W(CO)_6$ has been predicted by state-of-the-art theoretical calculations, which predict a decreasing trend in bond strength from $W(CO)_6$ to $Sg(CO)_6$ due to the relativistic destabilization of the 6d atomic orbitals in Sg.

B. Originality and significance:

The experimental set-up is innovative and the combination of two detectors, an isothermal section and a gradient section indeed allows a relative evaluation of first bond dissociation energies of $W(CO)_6$ and $Sg(CO)_6$.

C. Data & methodology:

The critical points raised in the ensuing discussion will most likely not change the main outcomes of the submitted paper. However, in view of a thorough scientific discussion there are few points that need to be addressed.

A crucial quantity is the ratio of volatile to non-volatile fractions, i.e. the ratio of count rates in the miniCOMPACT and the COMPACT detector. This ratio depends, strictly speaking, on several variables: the first bond dissociation energy (FBDE), the gas low rate, the temperature, the nuclear half-live of the investigated species, and the volatility of the investigated species.

Furthermore, the authors assume, that in the miniCOMPACT only non-volatile species i.e. $M(CO)_n$ species with M = W, Sg and n = 0…5 are detected, while in COMPACT only $M(CO)_6$ is detected. This is not entirely correct. It is conceivable, that $M(CO)_6$ species are also registered in the miniCOMPACT, since this species can a) decay radioactively in flight or b) decay radioactively while adsorbed on the surface. Also volatile species migrate slower through an open column than the carrier gas as their interaction with the surface is significantly stronger than for an atom of the carrier gas. The inner volume of the miniCOMPACT detector is 0.96 cm^3, so the carrier gas at a flow rate of 2.5 L/min passes through the miniCOMPACT in 23 ms on average, which is relatively short compared to the half-lives of $^{259g,m}Sg$ of 402 and 226 ms. The connecting capillary between miniCOMPACT

and COMPACT has a similar volume of 0.94 cm^3. By using the Monte Carlo model and the previously determined adsorption enthalpy of $M(CO)_6$ (M = W, Sg) on SiO_2-surface the fraction of $M(CO)_6$ detected in the miniCOMPACT could be calculated and shown to be negligible, but this discussion is missing.

Answer:

Thank you for this point. We did simulate the distribution of $W(CO)_6$ and $Sg(CO)_6$ in the whole detection system. Based on the distributions obtained by the MC simulations one can estimate the probability of decay-in-flight inside the miniCOMPACT for ^{259}Sg (~16%). For three ^{259}Sg events detected in the second COMPACT detector, this results in calculated 0.58 decay events of ^{259}Sg in the miniCOMPACT. The experimental distribution of ^{259}Sg events in the miniCOMPACT supports this. One decay from ^{259}Sg was registered in the second half of the detector array, whereas more than 70 ^{259}Sg events were registered in the first half of miniCOMPACT and assigned to non-volatile products. This one event may be from $Sg(CO)_6$ or still belong to the exponential distribution of the non-volatile fraction. By subtracting one event (as decay in flight) from the 72 events from ^{259}Sg observed in the miniCOMPACT detector, the ratio between the volatile and non-volatile fractions will change to 4:75 = 0.0533. However, this value is well inside the determined error bars for the ratio of volatile and non-volatile fractions, $0.04_{-0.02}^{+0.05}$, which was determined based on the Poisson statistics for uncertainty quantification for small event numbers, i.e., three events observed in COMPACT. The statistical uncertainties for the three ^{259}Sg events observed in COMPACT cover a rather large range of events in COMPACT, from 1.43 to 6.27, at the 1-sigma 68% confidence level, and thus result in large error bars for the ratio. Thus, statistical uncertainties also cover the uncertainty for the decay in flight in miniCOMPACT. Nevertheless, the difference (FBDE(W) – FBDE(Sg) = 4(2) kJ/mol was determined using the ratio value of $0.04_{-0.02}^{+0.05}$ giving the error bars of ± 2 kJ/mol, which are significantly smaller than in the experimental FBDE(W) value of 192(8) kJ/mol. The error bars of the experimental FBDE value of W provided the dominant contribution to the error bar of the FBDE value of Sg, 188(9) kJ/mol.

We have added additional explanations into the last paragraph of the Results section of the revised manuscript:

"Based on Monte Carlo simulations (described in the following section), the probability for decay in flight of volatile $^{259m,g}Sg(CO)_6$ was estimated (~16%) to be less than one event out of 72 ^{259m,g}Sg events registered in miniCOMPACT. This would change the ratio of volatile and non-volatile fractions to 0.05, which lies within the statistical error bars of the value $0.04_{-0.02}^{+0.05}$."

The issue of different half-lives of ^{259g}Sg and ^{259m}Sg and ^{161}W and ^{162}W needs to be addressed. Actually, the half-lives of ^{161}W and ^{162}W have not been mentioned at all. These are $T_{1/2}(^{161}W)$ = 409(18) ms and $T_{1/2}(^{162}W)$ = 1.19(12) s. The half-life of the nuclide is an important parameter in the Monte Carlo modeling of the migration of a $M(CO)_6$ molecule through miniCOMPACT and COMPACT.

Answer:

Thank you for this comment. Different half-lives of the W isotopes were taken into account, as it is described below. We added the half-lives for W isotopes to the text in the Methods section.

The authors did not mention what the production ratio of $^{161}W/^{162}W$ is, or what half-life they used in the Monte Carlo model. The half-lives differ by almost a factor of 3.

Answer:

The ratio of the produced W isotopes was experimentally determined from the alpha spectrum in the first detector of miniCOMPACT, where the activity is the highest. From the analysis of the alpha spectrum, the production ratio of ^{161}W:^{162}W is estimated to be 15:85 (s. Fig. 1 below). However, the alpha energy range used for the experimental distribution of both ^{18}W isotopes does not cover the low-energy tail of the alpha peaks. Therefore, the ratio of ^{161}W:^{162}W for the area highlighted in yellow in Fig. 1 is corrected to 25:75 and used for MC simulations.

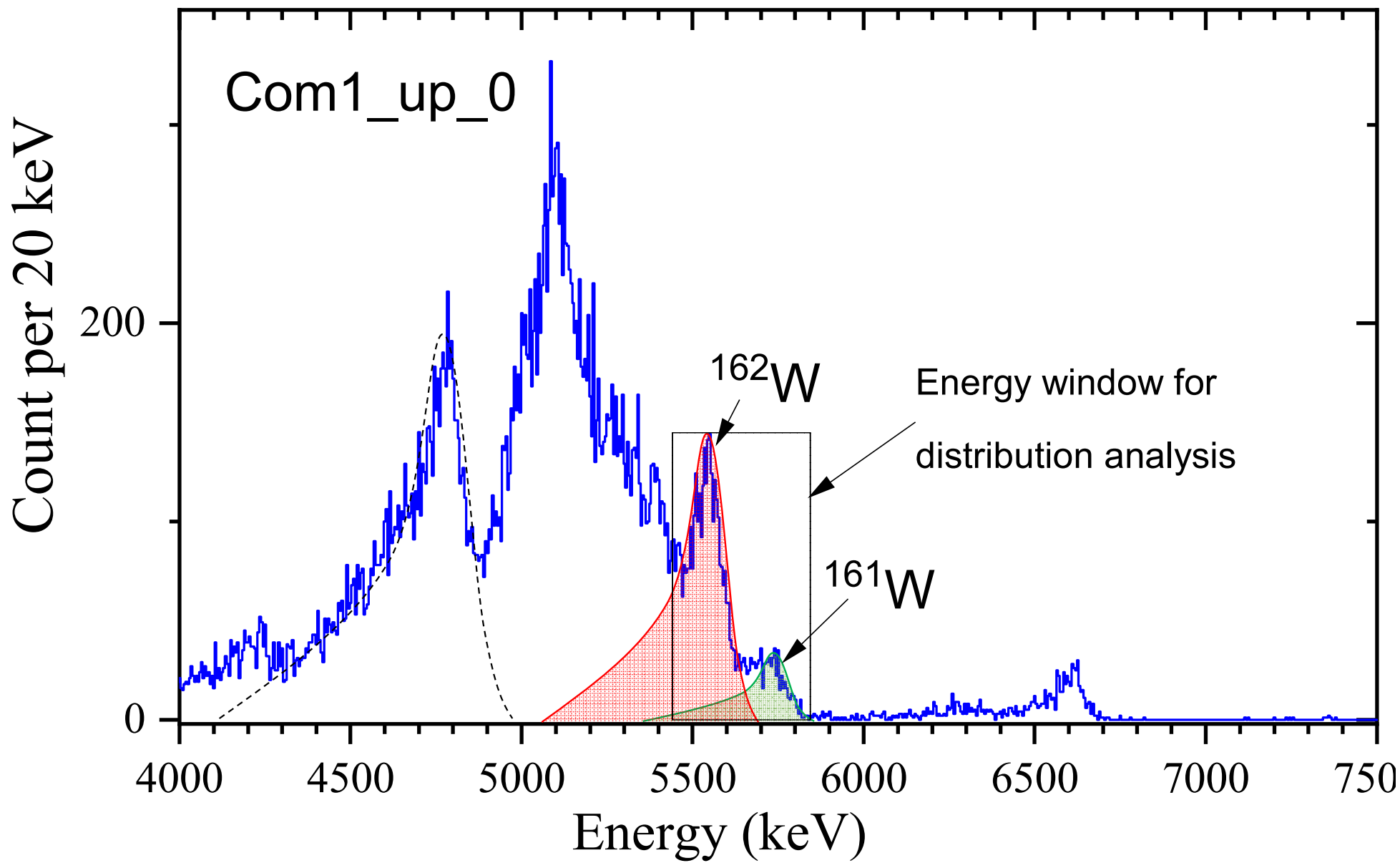

Fig. 1. Alpha spectrum from the first strip in miniCOMPACT. The alpha lines from 161,162W were identified among the decay products and products of other evaporation channels.

The MC simulations were performed for both W isotopes individually. The final distribution for both isotopes, ^{161}W and ^{162}W, was obtained by weighted summation. (s. Fig. 2).

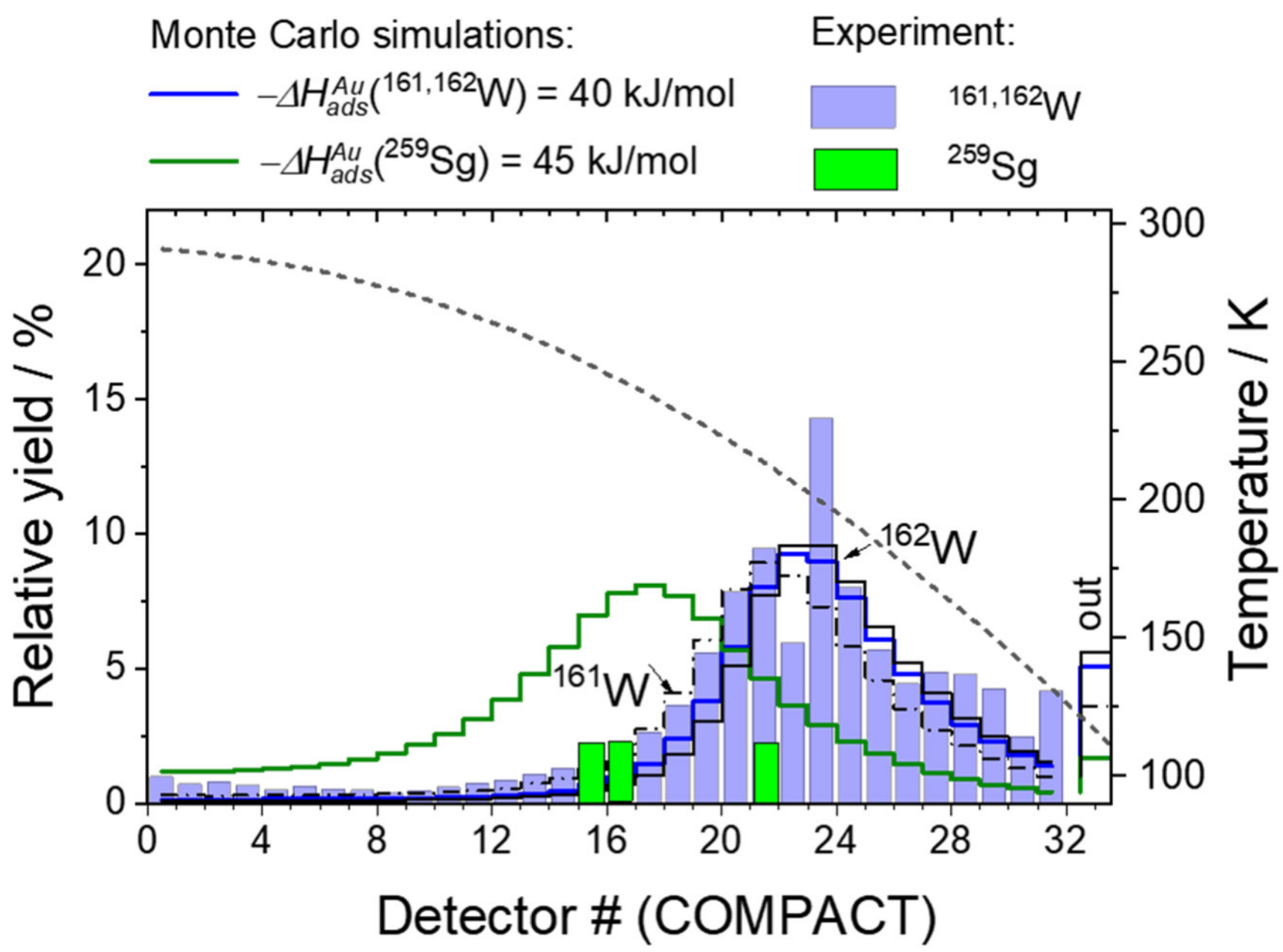

Fig. 2. The experimentally determined position of ^{161,162}W (blue bars) and ^{259}Sg (green bars) hexacarbonyl molecules observed in the COMPACT detector array and their distributions obtained in Monte Carlo simulations (blue and green lines, respectively). In addition, the simulated distributions for the individual ^{161}W and ^{162}W isotopes are shown as black lines.

The same applies to ^{259g}Sg to ^{259m}Sg, which according to Ref. 39 are both produced simultaneously and the half-lives differ by a factor of about 2.

Answer:

A similar procedure was also applied for the two states of ^{259}Sg. Alpha decays from two states in ^{259}Sg (ground and low-lying isomer) with slightly different half-lives (402(56) ms and 226(27) ms for the ground state (g) and metastable state (m), respectively) have been resolved in work by Antalic et al. thanks to a large statistics and conversion electron measurements (*Eur. Phys. J. A* **51**, 41 (2015)). It was found that both states are populated with similar probabilities, based on the analysis of similar numbers of alpha-decays with energies in the range of about 9.0-9.7 MeV. The half-life of these summed alpha events will be expressed by the weighted average value of the two half-lives, i.e., 314 ms. Such half-life value (320 ms) has experimentally been deduced by Folden et al. (Folden et al. *Phys. Rev. C* **79**, 027602 (2009)) prior to the work by Antalic et al., i.e., when alphas from different states were not resolved.

Actually, the authors were not able to assign whether the decays originated from ^{259g}Sg or ^{259m}Sg. The influence of different half-lives in determining the FBDE and also the adsorption enthalpies in the Monte Carlo modeling are small and probably negligible, but the authors should discuss this fact and provide arguments why this can be neglected.

Answer:

Thank you for the valuable remark. Actually, this issue was taken into account for W and Sg isotopes, but not discussed in the manuscript in detail. In the revised version we have added to the discussion the consideration about different MC distributions for isotopes/isomers with different half-lives:

"The ratio between two W isotopes with different half-lives was determined by the analysis of the alpha spectrum from the first strip of the miniCOMPACT detector array as ^{161}W:^{162}W = 25:75. The MC simulations of the distribution pattern were performed for the individual W isotopes. The final distribution for both isotopes, ^{161}W and ^{162}W, was obtained by weighted summation. For the simulation of the distribution pattern of ^{259}Sg, an averaged half-life value of 320 ms was used, as obtained by Folden et al. in the experimental study using the same nuclear reaction ^{52}Cr + ^{208}Pb. This value is in agreement with the averaged value for the two states $^{259m,g}Sg$ observed by Antalic et al."

One reference was added:

Folden III, C. M. et al. Measurement of the $^{208}Pb(^{52}Cr, n)^{259}Sg$ excitation function. *Phys. Rev. C* **79**, 027602 (2009) https://doi.org/10.1103/PhysRevC.79.027602.

From previous studies it is known that the adsorption enthalpies of group 6 hexacarbonyls on SiO_2 surface are very similar. It seems therefore justified to simply determine the ratio of volatile and non-volatile fractions, but it could be mentioned in one or two sentences, why this is the case.

Answer:

Thank you for the suggestion. We have added an additional explanation to the text in the Discussion section:

"Similarly low adsorption enthalpy values on the SiO_2 surface for both $W(CO)_6$ and $Sg(CO)_6$, determined in the previous study,(ref.11) allow for a rather simple ratio determination between the volatile ($M(CO)_6$) and non-volatile ($M(CO)_n$ ($1 \leq n \leq 5$; M = $^{161,162}W$, ^{259}Sg) fractions. The analysis of the simulated distributions in both detector arrays suggests that the probability for the decay in flight of ^{161}W, and especially ^{162}W, in the miniCOMPACT is low and can be statistically neglected. Thus, the ratio between the sums of $^{161,161}W$ and $^{259m,g}Sg$ events registered in COMPACT and miniCOMPACT, respectively, can be taken as the ratio between volatile and non-volatile fractions."

It appears from the presented data, that there were some minor issues in the operation of the miniCOMPACT and the COMPACT detector in experiments with $^{161,162}W$. It appears as if detector pair 15 in miniCOMPACT was not working, also it seems like only one side of detector pair 23 was operational in COMPACT.
Answer:

We added a sentence about the functionality of the detection arrays to the Methods section:

"The detector pair 15 of miniCOMPACT and the bottom strip 23 of COMPACT lost functionality during the experiment."
D. Appropriate use of statistics and treatment of uncertainties

From the manuscript it appears as if 1 sigma uncertaninties are used. This is especially important to mention when discussing the 3 events of Sg in the COMPACT detector.

Answer:

In the paragraph just before the Discussion section, we wrote "*Using Poisson statistics for uncertainty quantification, the three observed Sg events in COMPACT translate to 1.43 – 6.27 events at the 68% confidence level.*[41]", i.e., that we used 1 sigma (68%) uncertainty. For the small number of events, it translates to a rather large interval for Sg event number in COMPACT, from 1.43 to 6.27.

E. Conclusions: robustness, validity, reliability

In summary, the paper by Yakushev et al., demonstrates that on a one-atom-at-a-time level chemical experiments can be performed that allow to deduce the first bond dissociation energy of a molecule such as $Sg(CO)_6$ and that relativistic effects weaken the bond strength compared to the lighter homolog $W(CO)_6$.

F. Suggested improvements:

I recommend publication of the paper after the authors took care of the few points of criticism raised under C and D.

G. References:

All relevant references were cited.

H. Clarity and context:

The paper is well written and the provided graphs and drawings are of very good quality.

signed Prof. em. Dr. Andreas Türler

I appreciate the thoughtful responses to my comments and questions from the first round of review. In reading the comments of the other referees and responses from the authors of the manuscript, I am not yet convinced that a quantitative binding energy can be reported from a measurement that, as only plausibility arguments are made, is more qualitative in nature.

I have further comments and questions below.

On speciation:
In addressing my concerns regarding chemical speciation, the authors argue that the chemical community has not historically required direct speciation, citing past studies on hassium tetroxide and seaborgium hexacarbonyl. In the case of seaborgium hexacarbonyl, the chemical state was necessarily assumed and only indirectly verified by comparing its adsorption enthalpy on a quartz surface with theoretical predictions.

It is correct that the comparison was against theoretical calculations, but not only. The manuscript by Even et al. also reported the direct comparison of the experimental deposition of Sg with those of Mo and W.

However, more than 20 years after the initial chemical investigations of hassium, we must question whether the field should still accept inferred speciation as the standard, particularly as experiments become increasingly complex and a wider variety of assumed species are involved. Even if direct speciation is impossible for superheavy elements, modern techniques like time-of-flight mass spectrometry can identify the species produced for their higher-yield lighter homologues. If such direct speciation had been performed for the molecular tungsten species present in this study, the extrapolated assumptions regarding the chemical state of seaborgium would be significantly more convincing.

Thank you for this comment. After the first study of the Sg carbonyl complex, further investigations were performed, e.g., studies of carbonyl complex formation adsorption studies, and speciation studies. The latter were performed using the laser ablation method for the formation of carbonyl complexes of W, Re and Os, in combination with the time-of-flight mass spectrometry for their identification (Yang Wang et al. Phys. Chem. Chem. Phys., 2019, 21, 7147; doi: 10.1039/c8cp07844k). These studies concluded on fast formation of carbonyl complexes and on high stability of complexes, which fulfil the 18-electrons rule. So, the technique proposed by the referee was already applied for the identification of carbonyl complexes with lighter homologs and confirmed the formation of $W(CO)_6$ based on the observation of its molecular ion.
We have added to the manuscript (last paragraph on page 6) a new sentence and the reference.

"Over the last decade, preparations for further studies with SHE carbonyl complexes focused on a path towards the Bh carbonyl complex,[31,32,35] *and on determining the FBDE of the Sg hexacarbonyl complex relative to that of the complexes of the lighter homologs.*[32,33,36] ***The study of the chemical speciation of W carbonyl compounds was performed using the laser ablation method in combination with time-of-flight mass spectrometry, confirming the identity of the species as $W(CO)_6$.***[37] *Several theory studies….."*
Ref. 37. Wang, Y. et al. The study of rhenium pentacarbonyl complexes using single-atom chemistry in the gas phase. *Phys. Chem. Chem. Phys*. **21**, 7147(2019) https//doi.org/10.1039/c8cp07844k.

On extraction of the BDE:
For simplicity, let's assume all of the assumptions the manuscript makes on speciation is correct.
Here, the author's argue that they can extract the BDE by simply comparing the amount of fullycoordinated W(CO)6 and Sg(CO)6 detected on compact to everything else detected on minicompact. They take the ratio of these two detectors and then calculate the BDE for the Sg complex using equation 4, given that the BDE for the W complex has already been measured to be 192(8) kJ/mol.
The authors point to reference [19], stating that the "individual CO bond dissociation energies can be determined from a measurement of the individual fragment concentrations". However, in reading reference [19], it is stated in the conclusions that "It is premature to claim that individual CO bond dissociation energies can be determined unambiguously from these measurements".
I decided to test this by comparing the decomposition mass spectra for both W(CO)6 and its lighter homologue Mo(CO)6. The authors of this manuscript report the mass spectrum of W(CO)6 in figure 4 as "the abundances of individual fragments in the equilibrated sate reflect their thermochemical and kinetic behavior (line 309)". Below I give the same mass spectrum of W(CO)6 and also the mass spectrum of Mo(CO)6 from NIST.

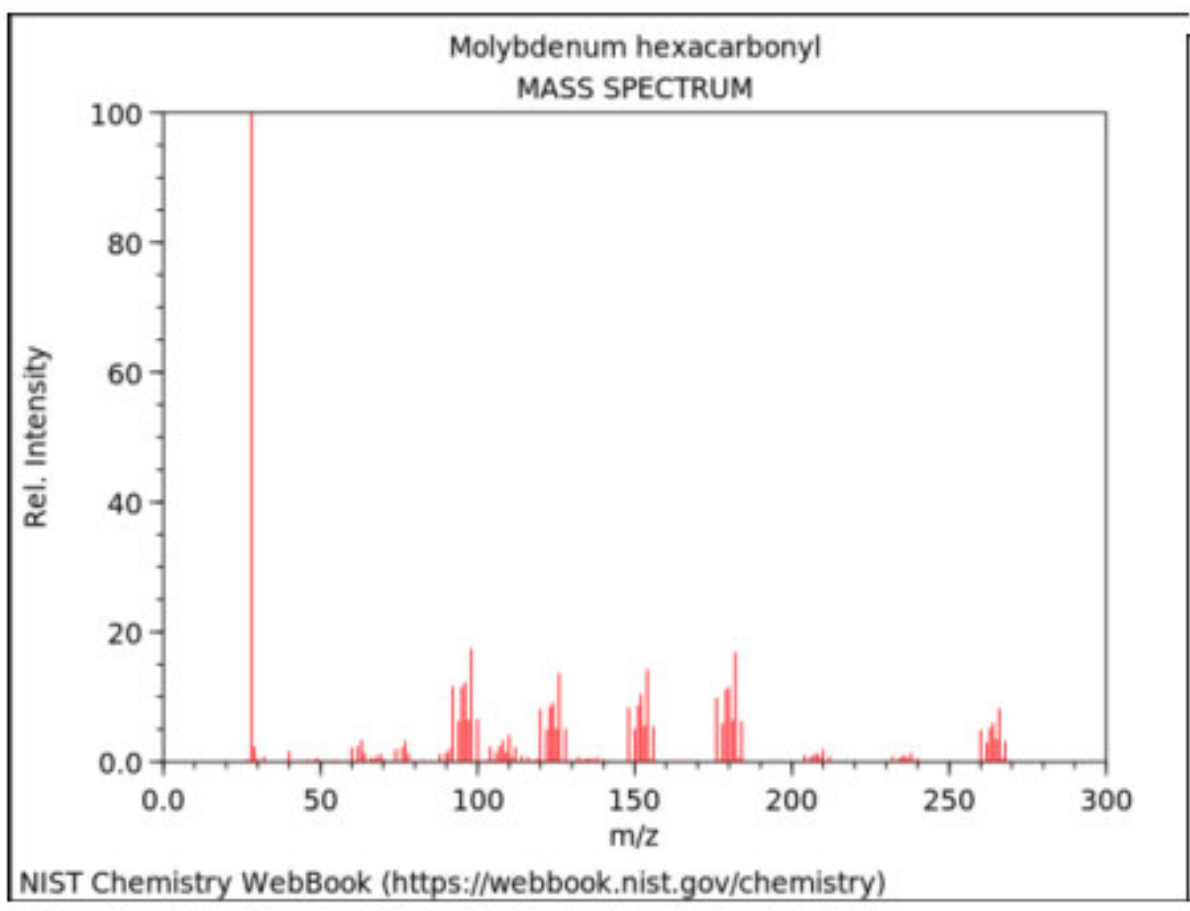

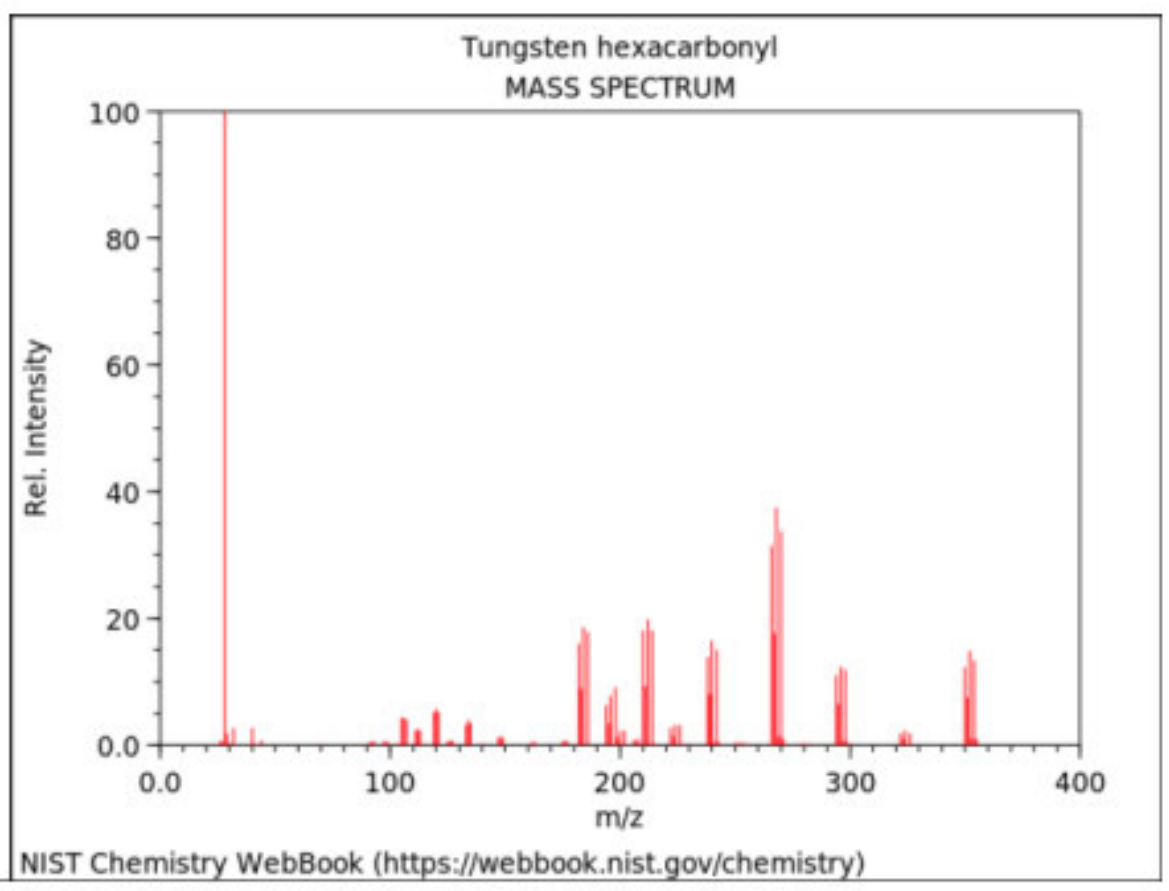

I compared the intensities from these spectra for the hexacarbonyl's versus the fragments and performed the same calculation from equation 4. My extracted intensities from these spectra are given in the table below. The BDE's for the sixth carbonyl of both of these species has been established, 192 for W and 169.45 for Mo. These are very different values, more different than what is being reported here for W and Sg. However, when I do this simple calculation, the ratio of (CO)6 to (CO)6-n for both is effectively the same, ~0.12. What am I missing here? Can the authors use these data to validate the method they claim?

Species	Mo	W
M	18	18
M(CO)	14	20
M(CO)2	14	17
M(CO)3	15	38
M(CO)4	2	14
M(CO)5	2	5
M(CO)6	8	15

Mo(CO)6 / Mo(CO)6-n = 8/65 = 0.12
W(CO)6 / W(CO)6-n = 15/112 = 0.13

The authors also confirm that it is expected for contaminant species and charged species to also be detected on the mini-compact detector. How does counting these species effectively as (CO)6-n species for the extraction of the FBDE not cause any issues?

Thank you for this comment. As discussed in our manuscript, the scheme shown in Fig. 4 confirms that species with higher abundance in the mass spectrum exhibit higher binding energy values. A direct comparison of fragment intensities from two different compounds can be performed - analogous to other, original studies, describing comparative dissociation studies of Mo and W carbonyl complexes via mass spectrometry under identical conditions.

As examples of such works with full description of the experimental procedure, which seems to be lacking for the NIST spectra, we provide the following references:

R. E. Winters and R. W. Kiser. Mass Spectrometric Studies of Chromium, Molybdenum, and Tungsten Hexacarbonyls. Inorg. Chem. 1965, 4, 2, 157–161; https://doi.org/10.1021/ic50024a006

G. D. Michels, G. D. Flesch, H. J. Svec. Comparative mass spectrometry of the Group 6B hexacarbonyls and pentacarbonyl thiocarbonyls. Inorg. Chem. 1980, 19, 2, 479–485; https://doi.org/10.1021/ic50204a039

Ref. 14 in our revised manuscript.

In these measurements, the intensities of $W(CO)_6$ in the mass spectra were always larger than those of $Mo(CO)_6$. To summarize, based on the comparative measurements of mass fragments, the FBDE values for Mo and W hexacarbonyl complexes were determined; the abundance and the FBDE value for the $W(CO)_6$ complex were consistently higher than that of the $Mo(CO)_6$ complex.

The referee's idea to compare the intensities of individual species from two different mass spectra, which are not normalized, is a bit surprising for us. As the table produced by the referee indicates, the sums of the intensities of all carbonyl fragments are different for these two spectra, and the warning statement in Ref. [19] fully applies to this situation. However, even such a simple comparison is indicative for a higher abundance for $W(CO)_6$ compared to $Mo(CO)_6$, as follows from the table prepared by the referee.

The authors comment that this result confirms "the trend of increasing FBDE values of hexacarbonyls in the group 6 is reversed from W to Sg (line 20)" and that this is "due to the
destabilization of the 6d atomic orbitals in Sg (line 23)". However, the reported value of 188(9)
for Sg is effectively the same value as the 192(8) for W. Given the error bars here, can such a
definitive statement be made?

Thank you for this comment. We have determined experimentally that the FBDE value for Sg is significantly smaller than that of W, by 4(2) kJ/mol, but our (as well as the referee's) emphasis is on the break of the trend established by three lighter homologs. The break of this trend cannot be extracted from comparing only the two values mentioned by the reviewer. As it is shown in the Fig. 5, the obtained FBDE value for Sg lies well below the expectation based on this increasing FBDE trend established by the values from the lighter homologs. Coming back to the second aspect, of the large uncertainties of the individual FBDE values, we write in the manuscript that these come from the large uncertainty of 8 kJ/mol of the experimental value of the FBDE value for $W(CO)_6$. Thus, our statement is correct.
